# The impact of extraction vs. non-extraction orthodontic treatment on the angulation of third molars on panoramic radiographs: a systematic review and meta-analysis

**DOI:** 10.1186/s40510-025-00573-3

**Published:** 2025-07-17

**Authors:** Nikolaos Kazanopoulos, Ioannis Xanthakis, Heleni Vastardis, Iosif Sifakakis, Dimitrios Konstantonis

**Affiliations:** 1 Department of Orthodontics, School of Dentistry, National and Kapodistrian University of Athens, Athens, Greece; 2 Department of Biostatistics and Health Data Science, Medical School-Department of Mathematics, National and Kapodistrian University of Athens, Athens, Greece

**Keywords:** Orthodontic treatment, Tooth extraction, Third molars, Molar angulation, Extraction vs non-extraction, Third molar eruption

## Abstract

**Background:**

The angulation of third molars is a critical factor influencing the likelihood of impaction. Orthodontic premolar extractions have been hypothesized to affect the eruption path of developing third molars by modifying available space and mesial drift patterns.

**Objective:**

This systematic review and meta-analysis aimed to assess whether premolar extraction during orthodontic treatment alters the angulation of developing third molars compared to non-extraction protocols.

**Eligibility criteria:**

Observational studies comparing angular measurements of third molars between extraction and non-extraction orthodontic treatments were included. Studies without a control group or adequate cephalometric data were excluded.

**Information sources:**

A comprehensive literature search was conducted across two electronic databases (MEDLINE, Scopus) up to November 2024, following PRISMA 2020 guidelines.

**Risk of bias and synthesis of results:**

Risk of bias was evaluated independently by two reviewers using the ROBINS-I tool. A random-effects meta-analysis was conducted, and the certainty of the evidence was assessed using the GRADE approach.

**Results:**

Nine studies (865 participants) were included. For mandibular third molars, extraction was significantly associated with improved angulation (SMD = − 0.37; 95% CI: − 0.59 to − 0.15; *p* = 0.004). Significant differences were found in M3L/M2L (MD = − 1.31; 95% CI: − 1.76 to − 0.85; *p* = 0.003) and M3L/PP (MD = − 4.85; 95% CI: − 8.50 to − 1.21; *p* = 0.02). No statistically significant difference was observed in the M3L/MP angle. In the maxilla, only the M3U–PP angle showed a significant change (MD = − 5.79; 95% CI: − 11.53 to − 0.04; *p* = 0.049). Meta-regression revealed no association with age, sex, or premolar type. Certainty of evidence ranged from low to moderate.

**Supplementary Information:**

The online version contains supplementary material available at 10.1186/s40510-025-00573-3.

## Introduction

The third molar has the highest impaction rate among all teeth, with a global prevalence which varies between 16.7% and 68.6% [1–9]. Most research indicates that there is no significant difference in third molar impaction between sexes [2, 3, 5, 7]. However, some studies have found a greater incidence in females compared to males [9, 10].

Third molar impaction is more commonly found in the mandible compared to the maxilla [11, 12]. Obstruction of mandibular third molar eruption can occur due to limited bone remodeling at the anterior part of the mandibular ramus [13]. Similarly, insufficient compensatory periosteal apposition along the posterior outline of the maxillary tuberosities may impede the eruption of maxillary third molars [14, 15]. Furthermore, in both jaws the eruption space available for third molars is affected by the direction of eruption of the posterior teeth [13, 16].

Third molars exhibit significant variability in size, shape, timing of mineralization, position, and eruption pathway [11]. Common complications associated with unerupted third molars include pericoronitis, root resorption of the second molar, periodontal disease, infections, and cyst formation [17]. Therefore, clinicians often advise patients for preventive removal of the third molars even when no apparent issues are present [18]. On the other hand, others argue that this approach may pose unnecessary surgical risks [19].

Many studies have suggested that orthodontic treatment involving the extraction of permanent teeth, such as the first or second premolars in the maxilla and/or mandible, may improve the eruption possibilities of the third molars [20, 21]. Orthodontic extraction treatments can induce mesial movement of first and second molars, thereby increasing the retromolar space [22, 23]. However, it is still unclear whether this increased space actually affects the possibility of third molar eruption [22–26]. Some studies suggest that non-extraction treatment leads to a higher incidence of third molar impaction [22, 24–26] while others show only minor differences between extraction and non-extraction cases [27]. Still, research on the effects of extraction therapy on maxillary third molar impaction is scarce. The positional relationship between the teeth selected for extraction during orthodontic treatment and the third molars has been suggested as a potential predictor of third molar eruption [28]. However, there is currently no evidence comparing the effects of first versus second premolar extraction on the angulation changes of third molars. Also, factors like bony base growth potential, amount of dental crowding, incisor angulation, and the implemented biomechanics may play a crucial role in determining the overall impact of extraction therapy on third molars [29]. In the mandible, these factors appear to have an even greater impact, as mesial molar movement and anterior eruption patterns of the posterior teeth can significantly increase retromolar space, thereby reducing the likelihood of impaction [13, 16, 28]. The angulation of third molars may also influence the probability of their eruption, with more upright orientation being considered more favorable [30].

It was therefore the purpose of this systematic review to investigate the angulation changes of third molars after orthodontic treatment between extraction and non-extraction groups of patients.

## Materials and methods

The protocol for this review was developed according the PRISMA 2020 guidelines (Preferred Reporting Items for Systematic Reviews and Meta-Analyses) (Appendix S1) and was registered in PROSPERO (CRD42024588516) [31].

### Information sources and search strategy

Between November 1 and November 7, 2024, we conducted a systematic literature search in MEDLINE and Scopus for studies published since January 1, 2000 (Appendix S2). Only full-text articles in English were considered. Keywords related to orthodontic treatment; tooth extraction and angulation of third molars were used. Additionally, all studies referenced in previous systematic reviews on tooth extractions and third molar angulation were screened for eligibility. Exploratory searches were also performed in Google Scholar and Web of Science, but due to significant overlap with records retrieved from MEDLINE and Scopus, these databases were not included in the final search strategy.

### Eligibility criteria

The eligibility criteria were decided based on the PICOTS (Patient population, Intervention, Comparator, Outcome, Timing, Setting) system (Table S1). Research surveys which reported at least one of the following posttreatment third molar angulation assessments were included in our study:

- The angles formed by the axes of the third molars to the palatal plane.
- The angles formed by the axes of the third and second molars to the mandibular plane.
- The angles formed by the axes of the third and second molars to the interorbital plane.
- The angle formed by the axis of the third molar to the axis of the second molar.
- The distance of the retromolar space assessed from a distal point of the second molar to a point at the posterior part of the mandibular ramus.

In order to be included in this systematic review, studies had to compare third molar angulation between two groups: an extraction and a non-extraction group. The extraction group had to include patients with extractions of teeth other than third molars. Eligible patients were males and females with a full complement of teeth, excluding the third molars, with no history of cleft lip/palate, dentofacial deformities or syndrome. At least one third molar had to be present, and patients must have had no prior orthodontic treatment or intra- or extra- oral surgery. The treatment plan had to include at least either one tooth extraction or a non-extraction approach with no use of extraoral or temporary anchorage devices.

### Study collection

Duplicate records were removed using a reference manager, and all remaining publications identified through the literature search were imported into the Rayyan web-tool for initial screening. Two reviewers (N.K. and G.X.) independently screened titles and abstracts against the inclusion criteria. Full-text screening was subsequently performed by the same reviewers for studies that passed the initial screening stage. Any disagreements were resolved through discussion and consensus.

### Data collection

Data extraction included details on the data source, participant characteristics (inclusion and exclusion criteria, key demographics), study design and analysis (sample size, handling of missing data), and measures of third molar angulation.

A Google Form and spreadsheet were developed for data collection and were piloted on two eligible studies by all reviewers to ensure clarity and consistency. Two reviewers (N.K. and G.X.) independently conducted the data extraction, while a third reviewer (D.K.) cross-checked for discrepancies. Duplicate studies with identical or overlapping data sources were identified and excluded based on study institutions, authors, and study periods.

### Assessment of risk of bias in individual studies

The ROBINS-I tool was used to assess the risk of bias in the included non-randomized studies of interventions [32]. Each study was evaluated across the seven domains of the ROBINS-I tool, namely: bias due to confounding, bias in the selection of participants into the study, bias in the classification of interventions, bias due to deviations from intended interventions, bias due to missing data, bias in the measurement of outcomes, and bias in the selection of the reported result. The overall risk of bias for each study was determined according to the highest level of risk observed across these domains and categorized as low, moderate or serious based on established criteria. The assessments were performed independently by two reviewers, and any disagreements were resolved through discussion. A summary of the criteria used is provided in Table 1. The ROBVIS (Risk of Bias Visualization) tool was used to graphically present the results of the risk of bias assessment [33]. 

**Table 1:** Criteria adopted for risk of bias assessment using the ROBINS-I tool

Domains	Criteria
Bias due to confounding	Studies must account for baseline angulation of third molars, age, sex, and treatment mechanics as potential confounders.
Bias in classification of interventions	The classification of patients into extraction and non-extraction groups must be clearly reported and based on defined clinical criteria.
Bias in selection of participants into the study	Eligibility criteria must be clearly defined and consistent across groups. Diagnostic methods used to identify inclusion must be valid.
Bias due to deviations from intended interventions	Studies were evaluated for clarity and adherence to the planned orthodontic mechanics. Deviations from the protocol should be clearly reported.
Bias due to missing data	Loss to follow-up, absence of baseline or post-treatment measurements, or unaccounted dropouts were considered high risk.
Bias arising from measurement of the outcome	Third molar angulation must be measured with validated radiographic methods and standardized cephalometric analysis.
Bias in selection of reported results	Selective reporting was assumed when results on third molar angulation were expected but not fully presented or inconsistently reported.
Overall risk of bias	If any domain was assessed as high risk, the overall risk was classified as high. If at least one domain raised some concerns, but none were high, the overall risk was moderate. If all domains were low risk, the overall classification was low.

### Data synthesis

**Fig. 1:**
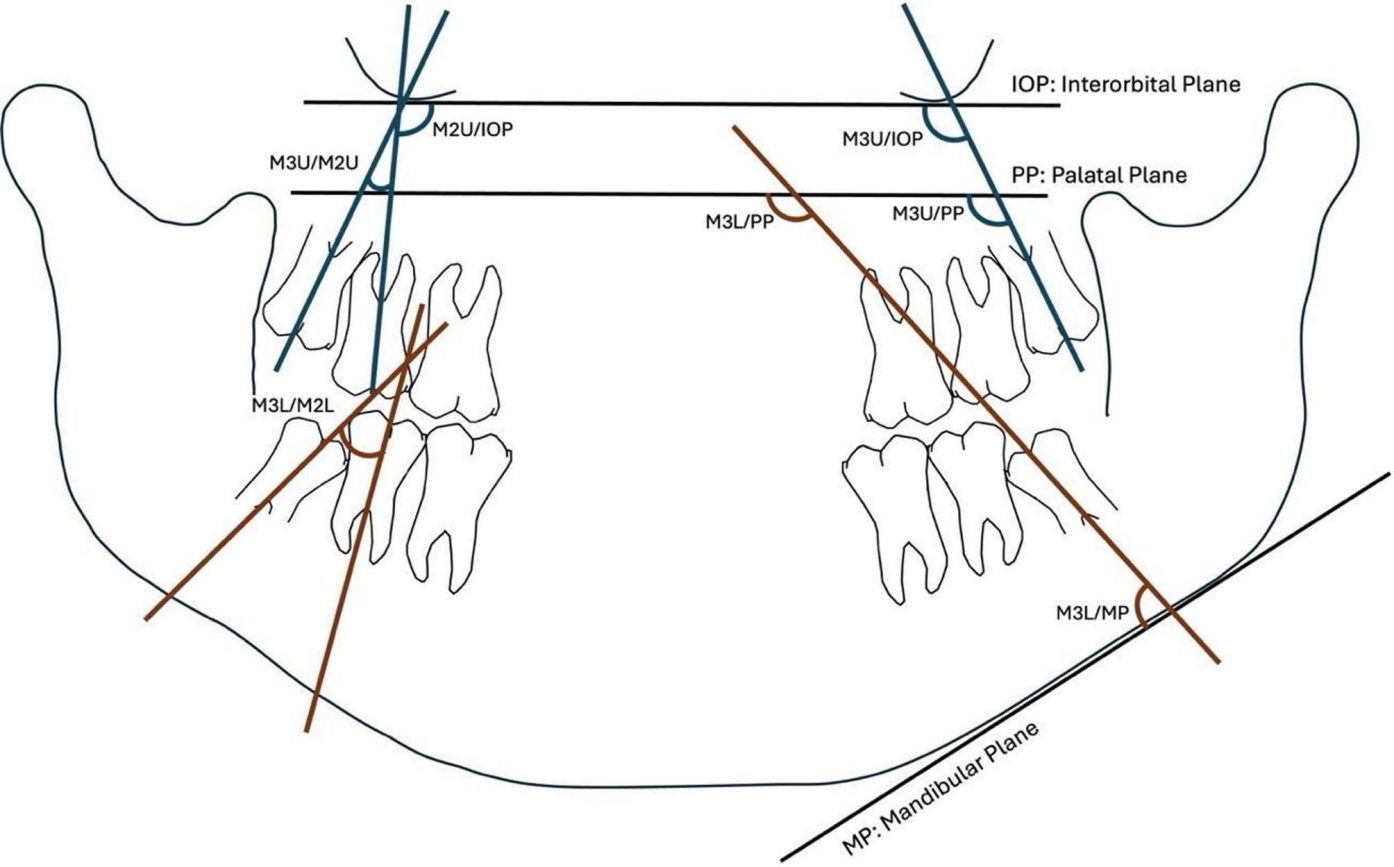
Schematic representation of the dental and skeletal reference planes and angular measurements used in the panoramic analysis. The interorbital plane (IOP), palatal plane (PP), and mandibular plane (MP) served as horizontal reference lines. The angular measurements M3U/IOP, M2U/IOP, M3U/PP, M3L/M2L, M3L/PP, and M3L/MP were those evaluated and meta-analyzed to assess the spatial positioning and angulation of maxillary and mandibular third molars

The primary outcome of the meta-analysis was the relationship between the angulation of third molars and the various planes or lines in panoramic radiographs. The following linear and angular measurements were considered (Fig. 1):

- The angle formed by the axes of the maxillary and mandibular third molars to the palatal plane (M3U/PP, M3L/PP).
- The angles formed by the axes of the maxillary and mandibular third and second molars to the mandibular plane (M3U/MP, M2U/MP, M3L/MP, M2L/MP).
- The angles formed by the axes of the maxillary and mandibular third and second molars to interorbital plane (M3U/IOP, M2U/ IOP, M3L/ IOP, M2L/ IOP).
- The angle formed by the axis of the third maxillary molar to the axis of the second maxillary molar (M3U/M2U).
- The angle formed by the axis of the third mandibular molar to the axis of the second mandibular molar (M3L/M2L).
- The distance of the retromolar space assessed from a distal point of the second molar to a point at the posterior part of the mandibular ramus as defined in the relevant investigations.

Some studies reported different outcome measures and, in some cases, only data for either the right or left side of the dentition were available. Considering the small number of studies and the large heterogeneity between them, a random-effects model was used to account for variability and Knapp-Hartung correction was used for the estimation of confidence intervals (CI). The Hedges’ G method and a restricted maximum likelihood estimator were applied to estimate tau-squared [34]. The difference used in the analysis was calculated as T1 (posttreatment) minus T0 (pretreatment), with measurements referring to the internal angulation of the third molar relative to the midline. For the meta-analysis, two pooled estimates were used. One was the Mean Differences (MD), meaning pooled estimates of the mean differences in the same angular measurement between pretreatment and posttreatment timepoints in the extraction and non-extraction groups. In order to be included in the analysis, angles would have to be reported in at least three studies. To account for studies contributing multiple effect estimates with a common control group and different intervention groups, we adjusted the effective sample size of the control group based on recommendations from the Cochrane Handbook (Sect. 23.1.4). In the literature, the intraclass correlation coefficient (ICC) varied from 0.6 to 0.8, but we preferred to use a more conservative ICC of 0.55 due to the small number of studies and the uncertainty in the accuracy of measurements.

Moreover, in cases where studies reported estimates separately for each side of the dentition, a sensitivity analysis was performed a priori by repeating the meta-analysis twice: once including only the right side and once including only the left side per study. The results were then compared with those obtained from the analysis where data from both sides were combined (Cochrane Handbook, Table 6.5.a). No significant differences were found, suggesting that including data from both sides did not substantially affect the overall findings; therefore, the combined effect measure was used as the primary analysis. Additionally, for studies that reported only baseline and post-treatment values without providing the mean differences and the corresponding standard deviations, we estimated the standard deviation of the change using the Cochrane formula for imputing standard deviations for changes from baseline (Sect. 6.5.2.8) [35].

The second pooled estimate was Standardized Mean Differences (SMD), which was used to compare the effect of extractions across different types of angular measurements. SMDs provided a more robust estimate considering the diversity of outcome measures across studies. Confidence intervals and prediction intervals were calculated, and results were presented using forest plots (Appendix S3).

### Meta-regression and subgroup analyses

To explore potential sources of heterogeneity among studies, meta-regression analyses were conducted using restricted maximum likelihood (REML) estimation. The following study-level covariates were tested independently: mean age of participants, percentage of female patients, and type of premolar extracted (first vs. second premolar). Meta-regression was performed separately for mandibular and maxillary third molar angulation changes. Covariates were included only when data were available from a sufficient number of studies to allow meaningful analysis. For maxillary third molars, meta-regression was based on MD since each angle was explored separately, while for mandibular third molars, SMD was used as the outcome measure since all mandibular angles were explored altogether.

On the other hand, a subgroup analysis was conducted to explore potential sources of heterogeneity and assess the impact of different outcomes on the pooled estimate. Specifically, studies involving mandibular extractions were analyzed, with the type of angulation serving as the independent variable. Three distinct angulation measurements were evaluated: M3L/PP, M3L/MP, and M3L/M2L.

### Sensitivity analysis

Sensitivity analysis was conducted [31] by applying the leave-one-out method to assess the robustness of the results against influential studies [36]. Publication bias was evaluated using funnel plots and Egger’s test [37]. All statistical analyses were performed using RStudio (version 2024.09.1) and R (version 4.4.2). A two-sided p-value ≤ 0.05 was considered statistically significant.

### Certainty of the evidence

The overall certainty of the evidence was assessed according to the Grading of Recommendations Assessment, Development, and Evaluation (GRADE) approach [38]. The evaluation was based on five domains: study design, risk of bias, inconsistency, indirectness, and imprecision. Each domain was rated as having no serious, serious, or very serious concerns. Since all included studies were observational, the initial certainty was considered low. The presence of large effects, consistency of results, and the absence of major sources of bias were considered for potential upgrading. The final certainty of evidence for each outcome was categorized as high, moderate, low, or very low. The GRADE assessment was performed manually by two reviewers, and disagreements were resolved through discussion.

## Results

### Study selection

A total of 349 articles were identified through the literature search, including 157 articles from Scopus and 192 from PubMed. After the removal of 131 duplicate entries, 218 articles remained for further evaluation. Title and abstract screening led to the exclusion of 172 articles, leaving 46 eligible for full-text assessment (Table S2).

**Fig. 2:**
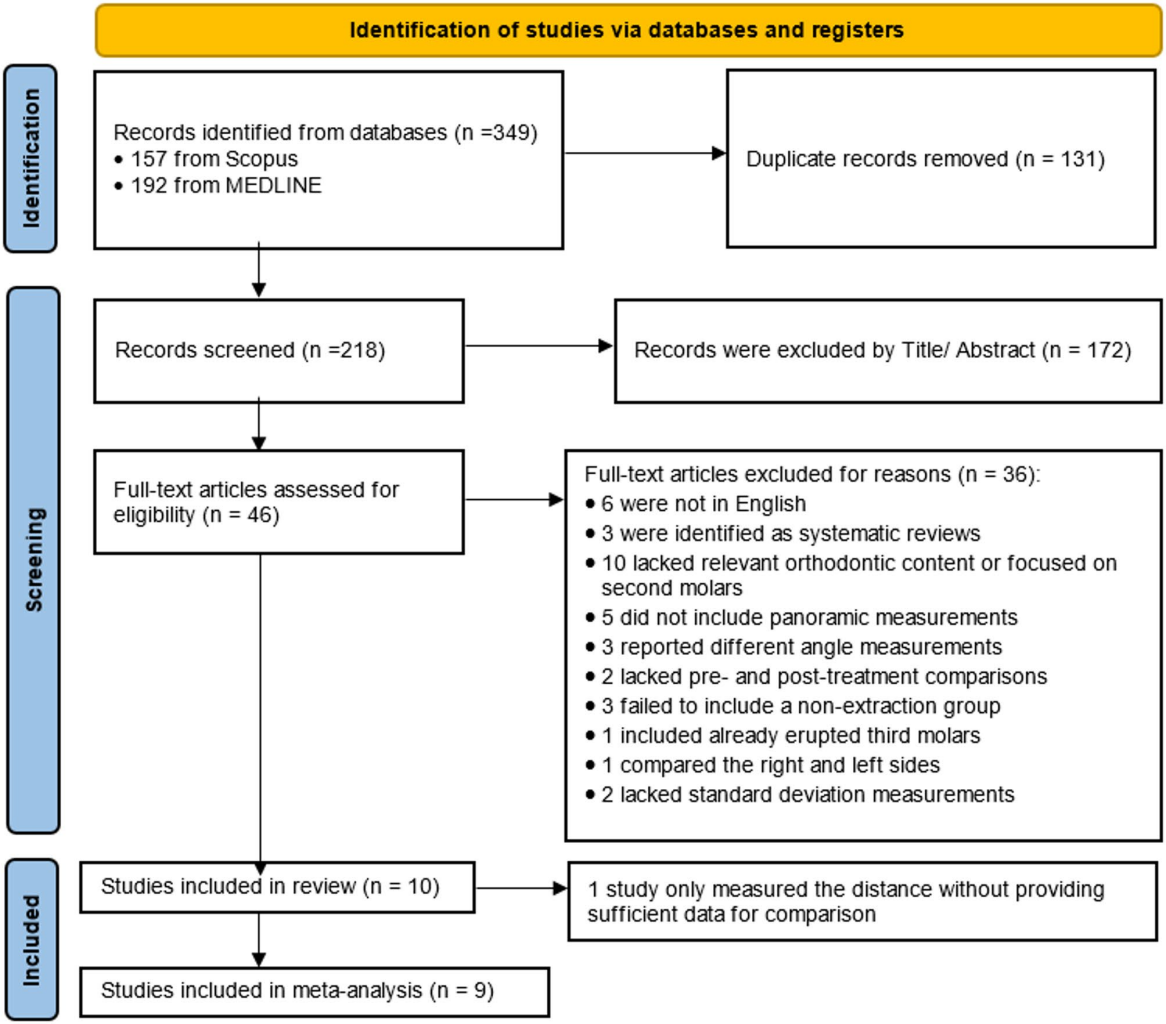
PRISMA flow diagram illustrating the identification, screening, eligibility assessment, and inclusion of studies in this systematic review and meta-analysis. Adapted from the PRISMA 2020 statement (Page MJ, et al., 2021)

During the full-text evaluation, 37 articles were excluded based on the following criteria: 6 articles were not published in English, while 3 were identified as systematic reviews. Additionally, 10 articles were excluded due to a lack of relevant orthodontic content or because they focused on second molars and 5 articles did not include panoramic measurements. Moreover, 3 articles reported different angle measurements, and 2 lacked pre- and post-treatment comparisons. Another 3 studies failed to include a non-extraction group, while 1 study involved already erupted third molars. Furthermore, 1 study compared the right and left sides, 2 articles lacked standard deviation measurements, and 1 study only measured the distance without providing sufficient data for comparison. After applying all eligibility criteria, 10 articles were included in the overall systematic review, while a total of 9 articles were deemed suitable and included in the meta-analysis. The study selection process is illustrated in the PRISMA flow diagram (Fig. 2), in accordance with PRISMA 2020 guidelines.

### Study characteristics

The characteristics of the nine studies included in the analysis are presented in Table 2. Out of these, seven studies (77.8%) were retrospective and two (22.2%) were prospective cohort studies. In total, 865 patients were included across all studies, with 471 in the extraction group and 394 in the non-extraction group. The sample sizes across the studies were relatively similar. The mean age of patients in the extraction group was 14.05 years, while in the non-extraction group it was 13.53 years. Regarding gender distribution, 53.64% of the patients were female and 46.46% were male.

**Table 2:** Characteristics of included studies

Study	Design	Extraction	Non-Extraction	Outcomes measured
de la Rosa et al. (2022) [11]	Retrospective	Sample: 116 (71 F/45 M), Age: 13.64, 1st Premolar *n* = 87 / 2nd premolar *n* = 29	Sample: 92 (50 F/42 M), Age: 13.20	M3U-PP, M3U-IOP, M2U-IOP
Singh et al. (2020) [39]	Prospective	Sample: 26 (10 F/16 M), Age: 20.46	Sample: 30 (15 F/15 M), Age: 20.51	M3L-M2L
Langer et al. (2023) [40]	Prospective	Sample: 39 (24 F/15 M), Age: 13.5, 1st Molar *n* = 39	Sample: 37 (23 F/14 M), Age: 14.5	M3U-PP, M3U-IOP, M2U-IOP, M3L-PP
Tarazona et al. (2010) [41]	Prospective	Sample: 58 (38 F/20 M), Age: 13.24, 1st Premolar *n* = 28 / 2nd premolar *n* = 30	Sample: 30 (18 F/12 M), Age: 13.24	M3L-MP
Jain and Valiathan (2009) [42]	Prospective	Sample: 25 (8 F/17 M), Age: 13.5, 1st Premolar *n* = 25	Sample: 25 (10 F/15 M), Age: 13.8	M3L-PP
Al Kuwari et al. (2013) [43]	Retrospective	Sample: 20 (10 F/10 M), Age: 12–15	Sample: 20 (10 F/10 M), Age: 12–15	M3L-PP
Durgesh et al. (2016) [44]	Prospective	Sample: 90 (45 F/45 M), Age: 13.67, 1st Premolar *n* = 90	Sample: 90 (45 F/45 M), Age: 13.41	M3L-M2L
Russell et al. (2013) [45]	Retrospective	Sample: 50 (25 F/25 M), Age: 13, 1st Premolar *n* = 25 / 2nd premolar *n* = 25	Sample: 24 (14 F/10 M), Age: 12.8	M3L-M2L
Peña-Reyes et al. (2024) [46]	Retrospective	Sample Class I: 23 (12 F/11 M), Age: 13.18, 1st Premolar *n* = 23 and sample Class II: 24 (11 F/13 M), Age: 12.84, 1st Premolar *n* = 24	Sample Class I: 23 (14 F/9 M), Age: 13.36 and Sample Class II: 23 (11 F/12 M), Age: 12.47	M3U-PP, M3L-PP

*F* Female, *M* Male

### Risk of bias

**Fig. 3:**
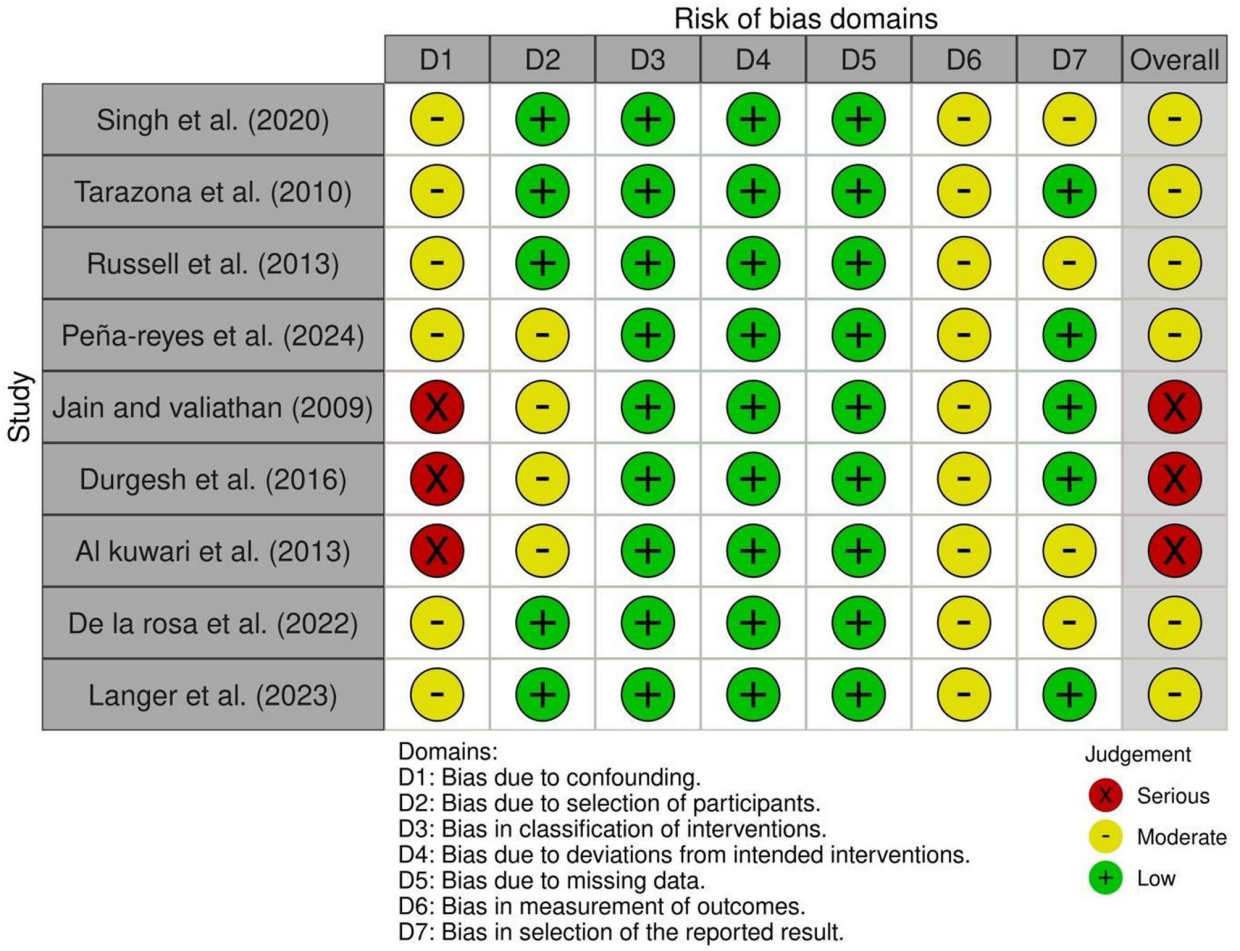
Risk of bias assessment using ROBINS-I tool

The risk of bias was assessed using the ROBINS-I tool. Among the nine non-randomized studies, the ROBINS-I tool was used to evaluate the risk of bias in the included non-randomized studies. The most frequent source of bias was inadequate control for confounding factors, such as baseline differences in skeletal class, sex, age, and growth stage, all of which may independently affect third molar angulation regardless of treatment. In particular, three studies (Jain and Valiathan [42]; Durgesh et al., [44]; Al Kuwari et al. [43]) were judged to have a serious risk of bias, mainly due to concerns in Domain 1 (confounding), limiting the validity of the comparisons between extraction and non-extraction groups. Additionally, moderate concerns were noted in the remaining six studies (Singh et al. [39]; Tarazona et al. [41]; Russell et al. [45]; Peña-Reyes et al. [46]; De la Rosa et al. [11]; Langer et al. [40]), primarily due to lack of blinding, incomplete outcome assessment, or unclear intervention classification. Although these studies were methodologically more robust, they still presented certain limitations, including inconsistent reporting and potential for residual confounding. A summary of the ROBINS-I evaluation is provided in Table 1, and the results are visualized using the ROBVIS tool in Fig. 3.

### Maxillary third molars

**Fig. 4:**
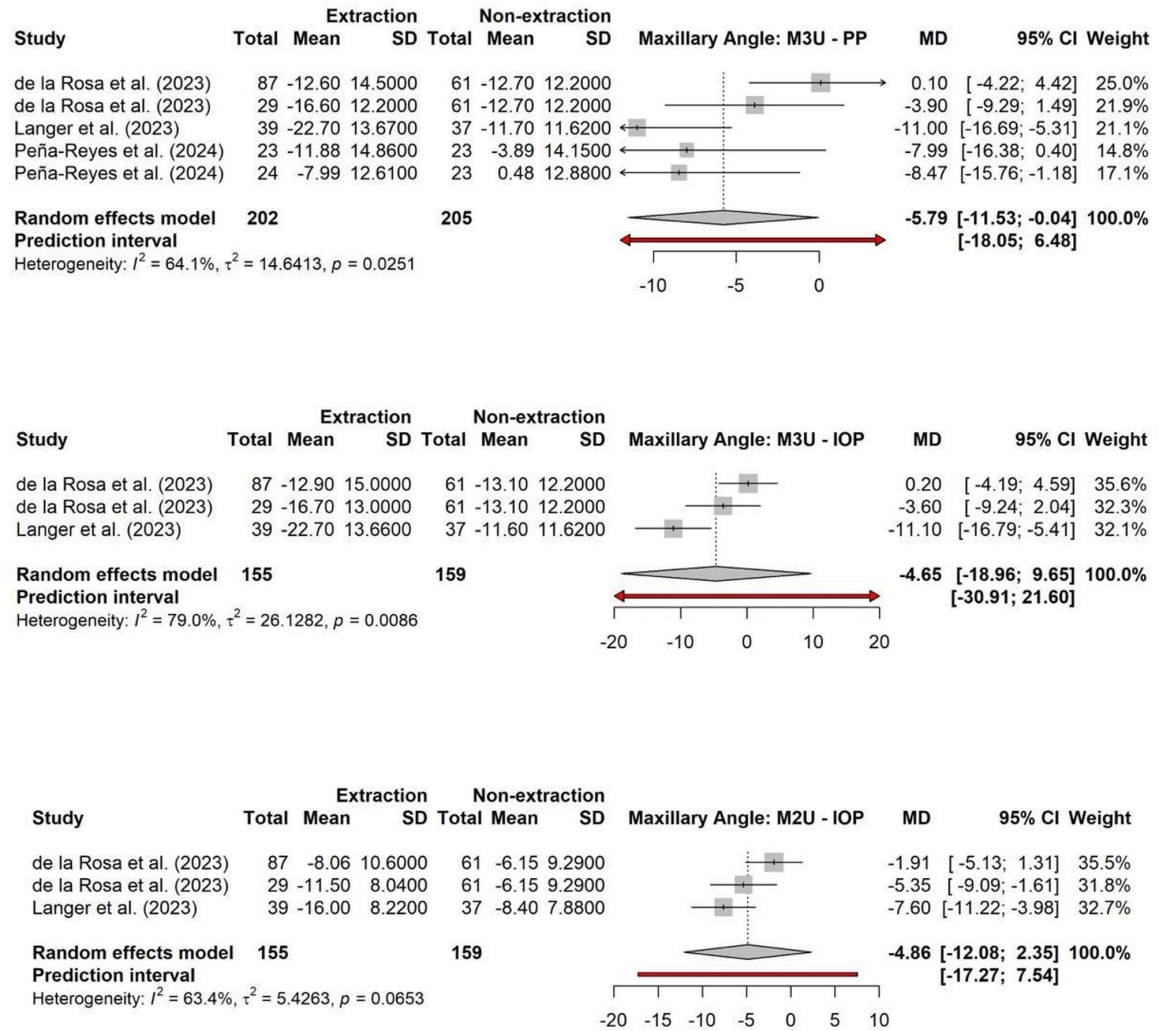
Forest plots of meta-analyses assessing maxillary third molar angular changes following orthodontic extraction versus non-extraction treatment across three angular measurements (M3U/PP, M3U/IOP, M2U/IOP). Pooled mean differences (MD) with 95% confidence intervals (CI) are shown for each outcome. Analyses were conducted using random-effects models and include prediction intervals and heterogeneity statistics

The three angles used to assess the angulation of the maxillary third molars were: M3U/PP, M3U/IOP, and M2U/IOP. The results of the meta-analysis, which compares the mean differences between T0 and T1 phases for both extraction and non-extraction groups, are detailed in Table 3. The maxillary angles were analyzed individually, with the mean differences (MDs) between the two groups combined as a pooled estimate. In all three angular measurements, the pooled MD was negative, indicating a reduction in third molar angulation from T0 to T1 in both groups (Fig. 4).

Specifically, of the three angles only the M3U/PP angle showed a statistically significant reduction between the extraction and non-extraction groups, with a pooled MD of -5.79 degrees (95% CI: -11.53 to -0.04; *p* = 0.049). The heterogeneity was substantial (I^2^ = 64.1%), and the Cochrane’s Q test approached significance (*p* = 0.03), which is reasonable considering the small number of included studies (Fig. 4). Although the M3U/IOP and M2U/IOP measurements were not statistically significant, the pooled mean differences indicated greater angular changes in the extraction group. Specifically, the M3U/IOP angle showed a pooled MD of -4.65 degrees (95% CI: -18.96 to 9.65; *p* = 0.30), while the M2U/IOP angle showed a pooled MD of -4.86 degrees (95% CI: -12.08 to 2.35; *p* = 0.10). Both outcomes exhibited substantial heterogeneity (I^2^ = 79% and 63.4%, respectively), which should be considered when interpreting the results (Fig. 4).

### Mandibular third molars

**Fig. 5:**
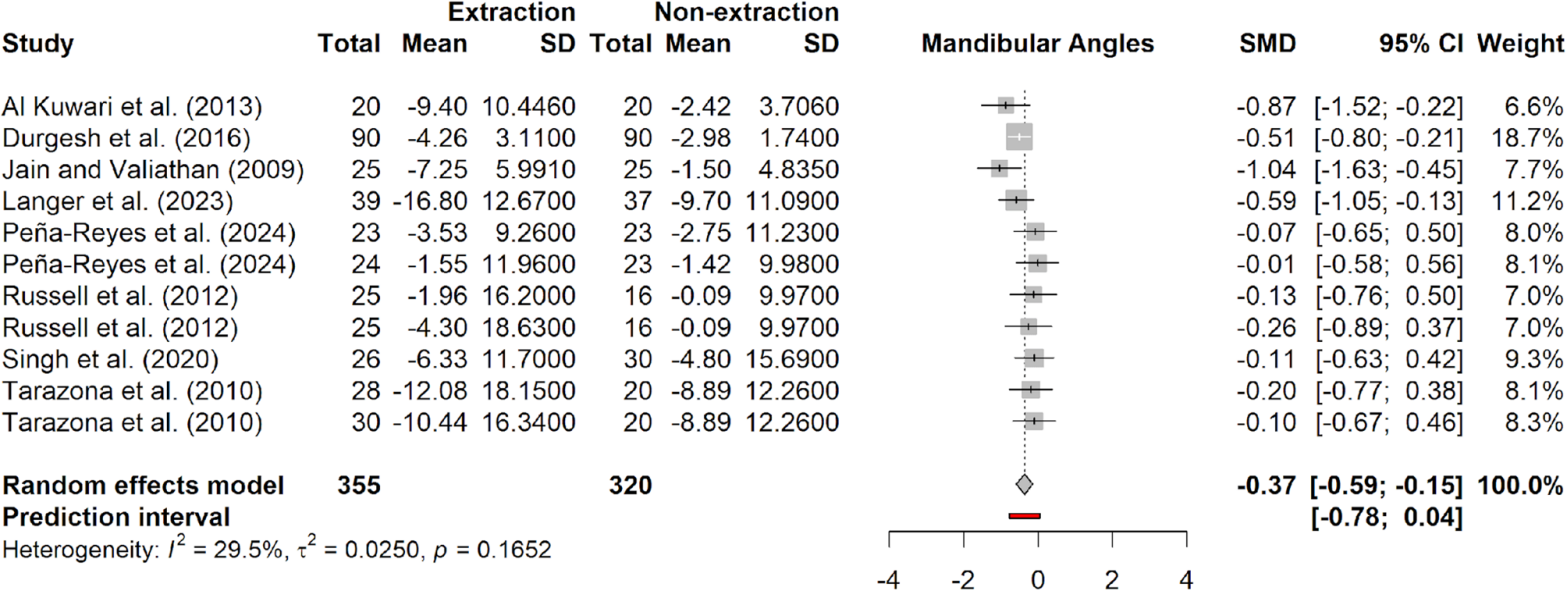
Forest plot of meta-analysis assessing mandibular third molar angular changes following orthodontic extraction versus non-extraction treatment. Pooled standardized mean difference (SMD) with 95% confidence intervals (CI) is shown. Analysis was performed using a random-effects model and includes prediction intervals and heterogeneity statistics

Regarding the mandibular third molars, three angles were analysed: M3L/M2L M3L/PP and M3L/MP. Three separate analyses were conducted using MD as the pooled estimate (Table 3). Two statistically significant differences were identified between the extraction and non-extraction groups in the M3L/M2L and M3L/PP angles. The pooled MD for M3L/M2L was − 1.31 degrees (95% CI: -1.76 to -0.85; *p* = 0.03), with minimal heterogeneity (I^2^ = 0%). However, most of the statistical weight in this analysis originated from a single study, while the others contributed with larger standard errors. For the M3L/PP angle, the pooled MD was − 4.85 degrees (95% CI: -8.5 to -1.2; *p* = 0.02), with moderate heterogeneity (I^2^ = 29.8%), and one study accounted for nearly half of the overall weight in the analysis. In the M3L/MP angle, the pooled MD was − 2.3 degrees (95% CI: -12.69 to 8.08; *p* = 0.22), with minimal heterogeneity (I^2^ =0%). To subdue potential heterogeneity and provide a more robust estimate, a comprehensive analysis was performed by pooling all mandibular outcomes using SMD. This robust analysis yielded an SMD of -0.37 degrees (95% CI: -0.59 to -0.15; *p* = 0.004), indicating a highly statistically significant difference between extraction and non-extraction groups. Heterogeneity was moderate (I^2^ = 29.5%), and Cochrane’s Q test approached significance (*p* = 0.17) (Fig. 5 and S1).

**Table 3:** Summary of findings table (SoF) for GRADE statement of included studies

Outcome	Mean difference (MD or SMD) [95% CI]	Participants (studies)	Certainty of evidence (GRADE)	Key message
M3L–M2L	MD: −1.31 [− 1.76, − 0.85], *p* = 0.03 *	318 (4 studies)	⊕⊕⊕⊖ Moderate^a^	There is moderate certainty that extraction results in a small but statistically significant reduction in the M3L–M2L angle.
M3L–PP	MD: −4.85 [− 8.50, − 1.21], *p* = 0.02 *	259 (5 studies)	⊕⊕⊖⊖ Low^ab^	There is low certainty that extraction results in a statistically significant reduction in the M3L–PP angle.
M3L–MP	MD: −2.30 [− 12.69, 8.08], *p* = 0.22	98 (2 studies)	⊕⊕⊖⊖ Low^ab^	There is low certainty about the effect of extraction on the M3L–MP angle, and the result is not statistically significant.
Overall mandibular angles	SMD: −0.37 [− 0.59, − 0.15], *p* = 0.004 *	675 (12 datasets from 9 studies)	⊕⊕⊕⊖ Moderate^b^	There is moderate certainty that extraction is associated with a small but statistically significant reduction in mandibular third molar angulation.
M3U–PP	MD: −5.79 [− 11.53, − 0.04], *p* = 0.049 *	407 (4 studies)	⊕⊕⊖⊖ Low^ab^	There is low certainty that extraction significantly reduces the M3U–PP angle.
M3U–IOP	MD: −4.65 [− 18.96, 9.65], *p* = 0.30	314 (3 studies)	⊕⊕⊖⊖ Low^ab^	There is low certainty about the effect of extraction on the M3U–IOP angle, with no statistically significant difference.
M2U–IOP	MD: −4.86 [− 12.08, 2.35], *p* = 0.10	314 (3 studies)	⊕⊕⊖⊖ Low^ab^	There is low certainty that extraction leads to a reduction in the M2U–IOP angle, but the effect was not statistically significant.

^a^Downgraded for imprecision due to wide confidence intervals

^b^Downgraded for inconsistency due to moderate to high heterogeneity (I^2^ >40%)

^c^Downgraded for both inconsistency and imprecision

^d^SMD used for outcomes with heterogeneous scales or units

^e^Statistically significant (*p* < 0.05)

### Sensitivity analysis

Sensitivity analyses were performed using the leave-one-out method. The pooled estimate remained nearly unchanged regardless of which study was excluded. In one instance, the statistical significance improved when a study that provided two estimates in the original analysis was removed, reducing heterogeneity. This finding suggests a lack of small study effects and indicates that larger studies did not disproportionately influence the pooled estimate. The use of a random-effects model ensured that all studies contributed similarly to the analysis (Table S3).

### Publication bias

Potential small study effects and publication bias were examined using a funnel plot and Egger’s test. Due to the small number of included studies, the funnel plot was not highly informative and did not exhibit a clear pattern. However, Egger’s test yielded a non-significant p-value, suggesting no strong evidence of publication bias (Figure S2).

### Meta-regressions and subgroup analyses

Meta-regression analyses were conducted to explore whether the percentage of female participants, mean age, or type of premolar extracted could explain heterogeneity among the included studies.

For the mandibular third molars, neither the percentage of female participants was significantly associated with the effect size (β = 0.0016, 95% CI: − 0.02 to 0.02, *p* = 0.86), nor was mean age (β = 0.01, 95% CI: − 0.21 to 0.23, *p* = 0.93). The type of premolar extracted also showed no significant association (Tooth PM2: β = 0.25, 95% CI: − 0.28 to 0.79, *p* = 0.30).

For the maxillary third molars, female percentage (β = 0.11, 95% CI: − 0.34 to 0.56, *p* = 0.50), mean age (β = − 2.14, 95% CI: − 14.56 to 10.28, *p* = 0.62), and premolar type (Tooth PM2: β = 0.02, 95% CI: − 1.77 to 1.81, *p* = 0.97) were not significantly associated with the standardized mean difference. Across all models, the proportion of residual heterogeneity explained (R^2^) ranged from 0 to 25.40%.

In addition to the meta-regression analyses, a subgroup analysis was conducted on mandibular third molar studies based on the type of angulation measured (M3L/PP, M3L/MP, and M3L/M2L). Pooled SMDs ranged from − 0.15 to − 0.51 across subgroups, with confidence intervals overlapping and no statistically significant difference detected between them (χ^2^ = 4.5, *p* = 0.1) (Figure S3). Heterogeneity varied across subgroups but remained generally low to moderate. Given the small number of studies within each group, this analysis was considered exploratory and should be interpreted with caution.

## Discussion

This study represents the first to our knowledge- systematic review and meta-analysis to investigate the effects of extraction and non-extraction orthodontic treatment on the angulation and eruption trajectory of the third molars. Specifically, our objective was to assess how orthodontic extraction therapy influences posttreatment third molar angulation, a parameter that may contribute to eruption potential. The results indicate significant differences in third molar angulation between the two different treatment modalities, providing valuable insights for orthodontic treatment planning.

### Mandibular third molars

The results of our study showed a highly statistically significant overall effect between extraction and non-extraction patients, with a SMD of -0.37 degrees. This finding suggests that extraction treatment is associated with a reduction in the angulation of third molars, as indicated by the negative SMD, thus increasing the possibilities for their eruption.

Our analysis also revealed distinct angular changes associated with extraction treatment across all the measured angles. Specifically, for the M3L/M2L and M3L/PP angles, the pooled MD were − 1.31 and − 4.85 degrees respectively indicating a statistically significant reduction in angulation in the extraction group compared to the non-extraction group. In contrast, the MD for the M3L/MP angle, was found to be decreased by 2.30 degrees but this decreasing trend was not statistically significant. These findings align with previous studies proposing that orthodontic extractions may facilitate mesial movement of posterior teeth, thereby increasing retromolar space and enhancing the conditions for third molar eruption [13, 36, 47]. Overall heterogeneity across and within studies was moderate at most. The included studies exhibited comparable effect sizes and similar sample sizes, which minimized the influence of any single study on the overall estimate. This robustness was further confirmed by the sensitivity analyses using the leave-one-out method, which yielded stable results regardless of the excluded study. Finally, the funnel plot didn’t reveal any obvious pattern between the studies pointing at the absence of publication bias and small study effects.

### Maxillary third molars

On the other hand, the effect on maxillary third molars remains less clear, as fewer studies have investigated this subgroup. The findings of this meta-analysis suggested a potential advantage of the extraction group over the non-extraction group; however, this conclusion is not supported by robust evidence. Specifically, although the M3U–PP angle demonstrated a statistically significant reduction in the extraction group, the M3U/IOP and M2U/IOP angles exhibited minor and non-significant changes. This may be partly attributed to the limited number of studies focusing on upper third molars and the significant heterogeneity observed across them. This limited and inconsistent evidence highlights a gap in the literature and underscores the need for further well-designed studies focusing on maxillary third molar angulation.

In our effort to explore potential sources of heterogeneity, we performed meta-regression analyses incorporating study-level covariates such as mean age, sex distribution, and type of premolar extracted. None of these moderators were statistically significantly associated with the treatment effect. Similarly, the subgroup analysis based on different angular measurements did not demonstrate significant between-group differences. While these findings suggest a degree of consistency across studies, they may also reflect limitations in the available data or insufficient power to detect true moderator effects. Future studies with individual participant data and standardized measurement protocols may help to better elucidate the factors influencing third molar angulation changes.

### Extraction vs. non extraction

When contemplating between extraction and non-extraction treatment like in borderline cases the clinician takes into consideration a series of parameters that influence the treatment decision. Specifically, issues like posttreatment stability, periodontal health, soft tissue changes and facial esthetics are among the key factors that can swing the pendulum towards one or the other treatment modality [16, 48–51]. Having evidence-based information on the factors influencing the eruption of third molars is a valuable resource for clinicians during patient consultation. This information can guide informed decision-making and help clinicians set realistic expectations regarding third molar eruption outcomes. While premolar extractions may offer advantages for the eruption of third molars, the decision should carefully weigh these benefits against the potential consequences. However, it should be noted that when the initial angulation of the third molar is extremely severe, even after premolar extractions and angular improvement, the chances of eruption may remain minimal [9, 52]. Therefore, the clinical benefit of improved angulation should be interpreted with caution in such cases.

Previous research suggests that premolar extractions may positively affect the available space and the vertical or angular positioning of third molars, potentially facilitating their eruption [12–14]. In line with these reports, the results of our meta-analysis endorse the hypothesis that orthodontic extractions can enable the angular correction of mandibular third molars. As third molar development and eruption are closely linked to the evolution of the dentition and the growth of the underlying skeleton, these results highlight important clinical considerations regarding the optimal timing of orthodontic treatment especially when extractions are involved [53, 54]. Undertaking an extraction treatment before growth peak, while the third molars are not yet fully developed, may improve their positioning and increase the likelihood of successful eruption [12]. However, angular correction alone may not guarantee eruption [22, 24–26], and future studies should investigate whether such changes actually lead to improved eruption outcomes.

With respect to measuring the distance in the retromolar space, our review identified only one study that met our inclusion criteria [30]. Bayram et al. [30] found that first molar extractions significantly increased the eruption space of third molars and decreased their impaction rate. Specifically, in the maxilla, 96% of third molars erupted in the extraction group compared to only 53% in the non-extraction group. In the mandible, 83% of third molars erupted in the extraction group, whereas 67% remained impacted in the non-extraction group. Moreover, third molar angulation improved significantly more in the extraction group, particularly in the maxilla. These findings suggest that first molar extractions can have a favorable impact on third molar positioning by providing additional space for eruption. Nevertheless, due to the presence of only this single study, a formal meta-analysis could not be performed.

Although both the PP and IOP were used as reference planes for angular measurement of maxillary third molars, we chose to analyze and report both due to their distinct anatomical and clinical implications. The palatal plane, while traditionally used in orthodontics, may be influenced by dental and skeletal changes during treatment. In contrast, the IOP is considered a more stable cranial reference, unaffected by dental movement. Therefore, including both angles allows for a more comprehensive and reliable assessment of third molar angulation [11, 55]. 

Our meta-analysis provides a quantitative synthesis of available data, demonstrating that premolar extractions are significantly associated with a reduction in third molar angulation. Despite some residual heterogeneity, the findings strengthen the conclusion that orthodontic extractions influence third molar positioning, thus contributing to the growing body of evidence on this topic. This research investigation further highlights the urgent need for more rigorous, well-designed, prospective studies to confirm these findings and refine clinical recommendations. Additionally, this systematic review and meta-analysis boasts several notable strengths; the protocol was pre-registered in PROSPERO [56], ensuring methodological transparency, and adhered to PRISMA guidelines. Advanced statistical methods, including random-effects models and sensitivity analyses, were employed to address variability across studies. The review also followed a meticulous and transparent process for data extraction and synthesis, with comprehensive documentation of the methodology and findings.

### Limitations

Nevertheless, certain limitations must be acknowledged. The included studies were predominantly retrospective in nature, which are inherently more susceptible to bias compared to prospective randomized controlled trials. The number of studies, mainly in the analysis of the upper extraction third molar angulation, was small and the total number of patients could ideally be larger. Additionally, while heterogeneity among the studies was addressed through subgroup and sensitivity analyses, significant variability remains, necessitating cautious interpretation of the results. This variability should be factored into clinical decisions to ensure the findings are appropriately applied to individualized orthodontic treatment planning. Angular measurements on panoramic radiographs are subject to distortion and positional variability, which may affect measurement accuracy and should be considered when interpreting the results. Moreover, most included studies used panoramic radiographs, which are known to have limitations such as lower resolution and geometric distortion; however, this modality remains widely used due to its lower radiation exposure compared to CBCT.

1. When a sample of orthodontic patients is morphologically homogenous then all changes detected at the end of treatment like any alterations in third molar angulation and position can be safely attributed to the treatment modality itself like extraction and non-extraction and not to preexisting differences.

Detailed reporting of patient characteristics, orthodontic appliances and mechanics used, as well as dental outcomes - such as changes in third molar angulation, eruption status, and available retromolar space -, should be a priority to enhance the validity of the findings. Furthermore, pre-registration of study protocols and the open sharing of measured data can significantly improve transparency, reproducibility, and the overall reliability of research findings.

## Conclusions

The results of this investigation showed that orthodontic treatment involving premolar extractions is significantly associated with angular improvement of mandibular third molars. The maxillary third molars although improved their angular position were found to be less affected by extraction treatment which, can be attributed to the limited number of studies that focus specifically on this group. By incorporating information about third molar eruption possibilities into both extraction and non-extraction treatment plans, clinicians can provide their patients with a more comprehensive approach to orthodontic care.

## Electronic supplementary material

Below is the link to the electronic supplementary material.

Supplementary Material 1

## Data Availability

No datasets were generated or analysed during the current study.

## Abbreviations

CI: Confidence interval
GRADE: Grading of recommendations, assessment, development, and evaluation
IOP: Infraorbital plane
MD: Mean difference
M3L: Mandibular third molar
M3U: Maxillary third molar
M2L: Mandibular second molar
M2U: Maxillary second molar
MP: Mandibular plane
PP: Palatal plane
PRISMA: Preferred reporting items for systematic reviews and meta-analyses
ROBINS-I: Risk of bias in non-randomized studies - of interventions
ROBVIS: Risk of bias visualisation
RoB: Risk of bias
RCT: Randomized controlled trial
SD: Standard deviation
SMD: Standardized mean difference
