# Supplementary material for: The impact of extraction vs. non-extraction orthodontic treatment on the angulation of third molars on panoramic radiographs: a systematic review and meta-analysis
Source: Prog Orthod. 2025 Jul 17;26:25. doi: 10.1186/s40510-025-00573-3 (PMC12270985; doi:10.1186/s40510-025-00573-3)
Supplement: Supplementary file 1 — Supplementary Material 1 [file 40510_2025_573_MOESM1_ESM.docx]

**Supplementary Materials**

**Table S1. List of inclusion and exclusion criteria (based on PICOTS).**

| Field | Inclusion | Exclusion |
| --- | --- | --- |
| Patients | Male and female patients of any age with Class I malocclusion and a full complement of teeth | Animal studies In vitro studies |
| Intervention | Extraction of teeth other than third molars | No extractions performed Third molar extraction only |
| Comparison | Patients undergoing non-extraction orthodontic treatment | – |
| Outcome | Angular measurements of third molars, including at least one of the following: • Third molar to palatal plane • Third molar to second molar axis • Third or second molar to mandibular plane • Third or second molar to Frankfort horizontal plane • Retromolar space distance | No angular or retromolar space measurements Inadequate data preventing quantitative synthesis |
| Study design | Randomized controlled trials (RCTs) or prospective/retrospective cohort studies | Studies older than 24 years (or data collected before 2000) Conference abstracts, case reports, reviews, editorials, protocols, guidelines non-English full text |

**Table S2: List of included and excluded studies, with the corresponding reasons.**

| Nr | Paper | Status |
| --- | --- | --- |
|  | Abu Aihaija ES, McSheny PF, Richardson A. A cephalometric study of the effect of extraction of lower first permanent molars. J Clin Pediatr Dent. 2000 Spring;24(3):195–8. | Excluded; non-eligible |
|  | Narula H, Goyal V, Verma KG, Jasuja P, Sukhija SJ, Kakkar A. A comparative evaluation of fractural strength and marginal discrepancy of direct composite veneers using four different tooth preparation techniques: An in vitro study. J Indian Soc Pedod Prev Dent. 2019 Mar;37(1):55–9. | Excluded by title/Abstract |
|  | Kateel SK, Agarwal A, Kharae G, Nautiyal VP, Jyoti A, Prasad PN. A Comparative Study of Canine Retraction by Distraction of the Periodontal Ligament and Dentoalveolar Distraction Methods. J Maxillofac Oral Surg. 2016 Jun;15(2):144–55. | Excluded by title/Abstract |
|  | da Costa Monini A, Júnior LGG, Vianna AP, Martins RP. A comparison of lower canine retraction and loss of anchorage between conventional and self-ligating brackets: a single-center randomized split-mouth controlled trial. Clin Oral Investig. 2017 Dec;21(4):1047–53. | Excluded by title/Abstract |
|  | Keng FY, Quick AN, Swain MV, Herbison P. A comparison of space closure rates between preactivated nickel-titanium and titanium-molybdenum alloy T-loops: a randomized controlled clinical trial. Eur J Orthod. 2012 Feb;34(1):33–8. | Excluded by title/Abstract |
|  | Alwafi AA, Hannam AG, Yen EH, Zou B. A new method assessing predicted and achieved mandibular tooth movement in adults treated with clear aligners using CBCT and individual crown superimposition. Sci Rep. 2023 Mar 11;13(1):4084. | Excluded by title/Abstarct |
|  | Su H, Xu K, Han B, Chen G, Xu T. A retrospective study of factors contributing to anchorage loss in upper premolar extraction cases. Niger J Clin Pract. 2022 Dec;25(5):664–9. | Excluded by title/Abstarct |
|  | Richardson G, Russell KA. A review of impacted permanent maxillary cuspids--diagnosis and prevention. J Can Dent Assoc. 2000 Oct;66(9):497–501. | Excluded by title/Abstarct |
|  | Georgalis K, Woods MG. A study of Class III treatment: orthodontic camouflage vs orthognathic surgery. Aust Orthod J. 2015 Nov;31(2):138–48. | Excluded by title/Abstarct |
|  | Currier GF, Kadioglu O. Alveolar bone changes in non-extraction orthodontics. In: Computed Tomography: New Research [Internet]. 2013. p. 299–311 | Excluded by title/Abstract |
|  | Hong SY, Shin JW, Hong C, Chan V, Baik UB, Kim YH, et al. Alveolar bone remodeling during maxillary incisor intrusion and retraction. Prog Orthod. 2019 Dec 23;20(1):47. | Excluded by title/Abstarct |
|  | Lee JE, Jung CY, Kim Y, Kook YA, Ko Y, Park JB. Analysis of Alveolar Bone Morphology of the Maxillary Central and Lateral Incisors with Normal Occlusion. Medicina (Kaunas). 2019 Sep 3;55(9). | Excluded by title/Abstarct |
|  | Palone M, Pignotti A, Morin E, Pancari C, Spedicato GA, Cremonini F, et al. Analysis of overcorrection to be included for planning clear aligner therapy: a retrospective study. Angle Orthod. 2023 Jan 1;93(1):11–8. | Excluded by title/Abstarct |
|  | Thilagalavanian A, Weir T, Meade MJ. Analysis of predicted and achieved root angulation changes in teeth adjacent to maxillary premolar extraction sites in patients treated with the Invisalign appliance. Am J Orthod Dentofacial Orthop. 2024 Nov;166(5):423–32. | Excluded by title/Abstract |
|  | Cellina M, Martinenghi C, De Nardi S, Palamenghi A, Cè M, Sforza C, et al. Anatomy of the Mental Foramen: Relationship among Different Metrical Parameters for Accurate Localization. Applied Sciences (Switzerland) [Internet]. 2023;13(16). | Excluded by title/Abstarct |
|  | Qiang R, Gao J, Wang Y, Wang W, Ma Y, Jin Z. Anchorage loss of the posterior teeth under different extraction patterns in maxillary and mandibular arches using clear aligner: a finite element study. BMC Oral Health. 2024 Oct 10;24(1):1204. | Excluded by title/Abstarct |
|  | de la Rosa MR, Langer LJ, Kouroupakis-Bakouros F, Jost-Brinkmann PG, Bartzela TN. Angular and positional changes of the maxillary third molars after orthodontic treatment with different premolar extraction patterns. Angle Orthod. 2022 Oct; | Included for outcome assessment |
|  | Gokalp H, Kaan Erdem M. Assessing position changes of impacted third molars in treatment of class II malocclusion with premolars extraction. Acta Odontol Scand. 2024 Oct 9;83:582–7. | Excluded; non-eligible |
|  | Knierim K, Roberts WE, Hartsfield JJ. Assessing treatment outcomes for a graduate orthodontics program: follow-up study for the classes of 2001-2003. Am J Orthod Dentofacial Orthop. 2006 Nov;130(5):648–55, 655.e1-3. | Excluded by title/Abstract |
|  | Golovcencu L, Anistoroaei D, Matei MN, Zegan G, Saveanu CI. Assessment of maxillary third molar status before and after orthodontic treatment in adolescent patients. Revista de Chimie. 2019;70(12):4381–6. | Excluded; non-eligible |
|  | Zhang Y, Cai P. Association between alveolar bone height changes in mandibular incisors and three-dimensional tooth movement in non-extraction orthodontic treatment with Invisalign. Orthod Craniofac Res. 2023 Feb;26(1):91–9. | Excluded by title/Abstract |
|  | Ponnada SR, Kantheti S, Gandikota C, Manne R. Beyond the ARCH: Examining mandibular third molar movements in response to premolar and single incisor extractions in orthodontic practice. Journal of Contemporary Orthodontics. 2024;8(2):215–21. | Excluded; non-eligible |
|  | Omori Y, Lang NP, Botticelli D, Papageorgiou SN, Baba S. Biological and mechanical complications of angulated abutments connected to fixed dental prostheses: A systematic review with meta-analysis. J Oral Rehabil. 2020 Jan;47(1):101–11. | Excluded by title/Abstarct |
|  | Sabbagh H, Khazaei Y, Baumert U, Hoffmann L, Wichelhaus A, Janjic Rankovic M. Bracket Transfer Accuracy with the Indirect Bonding Technique-A Systematic Review and Meta-Analysis. J Clin Med. 2022 Dec 4;11(9). | Excluded by title/Abstarct |
|  | Palone M, Fazio M, Pellitteri F, Guiducci D, Cremonini F, Pozzetti I, et al. CAD/CAM-based 3D-printed and PVS indirect bonding jig system accuracy: a systematic review, meta-analysis, and comparative analysis of hard and soft CAD/CAM transfer trays. Eur J Orthod. 2024 Jan 1;46(1):cjad069. | Excluded by title/Abstarct |
|  | da Costa Monini A, Júnior LGG, Martins RP, Vianna AP. Canine retraction and anchorage loss: self-ligating versus conventional brackets in a randomized split-mouth study. Angle Orthod. 2014 Sep;84(5):846–52. | Excluded by title/Abstarct |
|  | Duncan LO, Piedade L, Lekic M, Cunha RS, Wiltshire WA. Changes in mandibular incisor position and arch form resulting from Invisalign correction of the crowded dentition treated nonextraction. Angle Orthod. 2016 Jul;86(4):577–83. | Excluded by title/Abstarct |
|  | Singh S, Garg AK, Gupta DK, Singla L. Changes in mandibular third molar angulation in high-anchorage extraction vs non-extraction orthodontic treatment: A prospective study. J Contemp Dent Pract. 2020 Oct;21(10):1182–8. | Included for outcome assessment |
|  | Shrafeldin A, Abu Shahba R, Hafez H, Kader HMA. Changes in maxillary dental arch parameters concomitant to maxillary 1st premolar extraction in orthodontic treatment of angle class II dental malocclusion. International Journal of Clinical Dentistry. 2018;11(3):179–90. | Excluded by title/Abstarct |
|  | Burashed H. Changes in the Vertical Dimension After Orthodontic Treatment in Response to Different Premolar Extraction Patterns. Cureus. 2023 Dec;15(5):e38893. | Excluded by title/Abstarct |
|  | Janson G, F Almeida J, Valerio MV, Velásquez G, Aliaga-Del Castillo A, Gamba Garib D. Changes in third molar position after Class II subdivision malocclusion treatment with asymmetric extractions. Orthod Craniofac Res. 2022 May;25(2):226–33. | Excluded; non-eligible |
|  | Jang JM, Song JS, Lee JH, Choi HJ, Kim SO. Changing the Angulation of the Tooth Germ in the Bony Crypt: A Case Report. J Clin Pediatr Dent. 2018;42(5):401–5. | Excluded by title/Abstarct |
|  | Vilanova L, Henriques JFC, Patel MP, Reis RS, Grec RH da C, Aliaga-Del Castillo A, et al. Class II malocclusion treatment changes with the Jones jig, Distal jet and First Class appliances. J Appl Oral Sci. 2020;28:e20190364. | Excluded by title/Abstarct |
|  | Jiang T, Wu RY, Wang JK, Wang HH, Tang GH. Clear aligners for maxillary anterior en masse retraction: a 3D finite element study. Sci Rep. 2020 Jun 23;10(1):10156. | Excluded by title/Abstarct |
|  | Pour RD, Papageorgiou SN, Safi S, Eble OS, Jäger A, Gölz L. Clinical implementation of axial angulation of incisors in the course of routine fixed appliance treatment - a retrospective cohort study. Clin Oral Investig. 2023 Feb;27(2):659–69. | Excluded by title/Abstarct |
|  | Sridhara A, Konde S, Noojadi SR, Kumar NC, Belludi AC. Comparative Evaluation of Intraoral and Extraoral Periapical Radiographic Techniques in Determination of Working Length: An In Vivo Study. Int J Clin Pediatr Dent. 2020 Jun;13(3):211–6. | Excluded by title/Abstract |
|  | Feng X, Jiang Y, Zhu Y, Hu L, Wang J, Qi Y, et al. Comparison between the designed and achieved mesiodistal angulation of maxillary canines and posterior teeth and influencing factors: First premolar extraction treatment with clear aligners. Am J Orthod Dentofacial Orthop. 2022 Aug;162(2):e63–70. | Excluded by title/Abstract |
|  | Dai FF, Xu TM, Shu G. Comparison of achieved and predicted tooth movement of maxillary first molars and central incisors: First premolar extraction treatment with Invisalign. Angle Orthod. 2019 Sep;89(5):679–87. | Excluded by title/Abstract |
|  | Moyano J, Montagut D, Perera R, Fernández-Bozal J, Puigdollers A. Comparison of changes in the dental transverse and sagittal planes between patients treated with self-ligating and with conventional brackets. Dental Press J Orthod. 2020 Feb;25(1):47–55. | Excluded by title/Abstract |
|  | Akinci Cansunar H, Uysal T. Comparison of orthodontic treatment outcomes in nonextraction, 2 maxillary premolar extraction, and 4 premolar extraction protocols with the American Board of Orthodontics objective grading system. Am J Orthod Dentofacial Orthop. 2014 Dec;145(5):595–602. | Excluded by title/Abstarct |
|  | Jain N, Vichare G, Bhosale V. Comparison of retraction efficacy of titanium-molybdenum and titanium-niobium alloy wires – A prospective split-mouth study. APOS Trends in Orthodontics. 2022;12(4):245–51. | Excluded by title/Abstarct |
|  | Tao T, Liang H, Yan X, Fan Q, Jiang Q, Jian F, et al. Comparison of Root Parallelism in Extraction Cases Treated with Clear Aligners vs. Fixed Appliances. Applied Sciences (Switzerland) [Internet]. 2022;12(22). | Excluded by title/Abstract |
|  | Fontes FPH, Bellini-Pereira SA, Aliaga-Del-Castillo A, Patel MP, Freitas MR de, Henriques JFC, et al. Comparison of the dentoskeletal and soft tissue changes with the cervical headgear and Jones Jig followed by fixed appliances in Class II malocclusion patients: A retrospective study. Int Orthod. 2020 Sep;18(3):424–35. | Excluded by title/Abstarct |
|  | Cicek O, Yilmaz H, Demir Cicek B. Comparison of the Mesiodistal Angulations of Canine and Molar Teeth in Different Types of Orthodontic Malocclusions: A Retrospective Study. Diagnostics (Basel). 2023 Apr 5;13(7). | Excluded by title/Abstarct |
|  | Verma S, Kumar V, Verma RK, Singh SP. Comparison of torque control between two commonly used 0.018-inch pre-adjusted edgewise bracket systems in North Indian population: a retrospective study. Clinical and Investigative Orthodontics. 2023;82(1):1–6. | Excluded by title/Abstarct |
|  | Yao CCJ, Lai EHH, Chang JZC, Chen I, Chen YJ. Comparison of treatment outcomes between skeletal anchorage and extraoral anchorage in adults with maxillary dentoalveolar protrusion. Am J Orthod Dentofacial Orthop. 2008 Nov;134(5):615–24. | Excluded by title/Abstarct |
|  | Im J, Cha JY, Lee KJ, Yu HS, Hwang CJ. Comparison of virtual and manual tooth setups with digital and plaster models in extraction cases. Am J Orthod Dentofacial Orthop. 2014 Apr;145(4):434–42. | Excluded by title/Abstarct |
|  | Yamen T, Ulas O. Computed tomography evaluation of palatal form in the transversal and sagittal fabrication process and the effect of first molar and canine crown angulations. Advanced Composites Letters [Internet]. 2019;28. | Excluded by title/Abstract |
|  | Ihlis RL, Giovanos C, Liao H, Ring I, Malmgren O, Tsilingaridis G, et al. Cone beam computed tomography indications for interdisciplinary therapy planning of impacted canines. Oral Surg Oral Med Oral Pathol Oral Radiol. 2023 Jan;135(1):e1–9. | Excluded by title/Abstarct |
|  | Park M, Allareddy V, Atsawasuwan P, Lee MK, Lee KC. Consideration of root position in virtual tooth setup for extraction treatment: A comparative study of simulated and actual treatment results. Korean J Orthod. 2023 Jan 25;53(1):26–34. | Excluded by title/Abstarct |
|  | Salek F, El Idrissi I, El Alloussi M, Zaoui F, Azaroual MF. Corono-radicular dilaceration of a maxillary central incisor: A case report. Int Orthod. 2019 Sep;17(3):606–12. | Excluded by title/Abstarct |
|  | Li R, Zhu C, Chu F, Yu Q, Fan D, Ouyang N, et al. Deep learning for virtual orthodontic bracket removal: tool establishment and application. Clin Oral Investig. 2024 Jan 27;28(1):121. | Excluded by title/Abstract |
|  | Shalish M, Har-Zion G, Zini A, Harari D, Chaushu S. Deep submersion: severe phenotype of deciduous-molar infraocclusion with biological associations. Angle Orthod. 2014 Mar;84(2):292–6. | Excluded by title/Abstarct |
|  | Kharb S, Malhotra A, Batra P, Arora N, Singh AK. Diode Laser versus Conventional Surgical Circumferential Supracrestal Fiberotomy in Preventing Relapse of Orthodontically Derotated Teeth: A Randomised Control Trial. Turk J Orthod. 2023 Dec 29;36(4):224–30. | Excluded by title/Abstarct |
|  | Seo KW, Kwon SY, Kim KA, Park KH, Kim SH, Ahn HW, et al. Displacement pattern of the anterior segment using antero-posterior lingual retractor combined with a palatal plate. Korean J Orthod. 2015 Nov;45(6):289–98. | Excluded by title/Abstarct |
|  | McArdle LW, McDonald F, Jones J. Distal cervical caries in the mandibular second molar: an indication for the prophylactic removal of third molar teeth? Update. Br J Oral Maxillofac Surg. 2014 Feb;52(2):185–9. | Excluded by title/Abstract |
|  | Livas C, Delli K. Does orthodontic extraction treatment improve the angular position of third molars? A systematic review. J Oral Maxillofac Surg. 2017 Mar;75(3):475–83. | Excluded: systematic review |
|  | Alessandri Bonetti G, Incerti Parenti S, Zanarini M, Marini I. Double vs single primary teeth extraction approach as prevention of permanent maxillary canines ectopic eruption. Pediatr Dent. 2010 Oct;32(5):407–12. | Excluded by title/Abstarct |
|  | Hadler-Olsen S, Sjögren A, Steinnes J, Dubland M, Bolstad NL, Pirttiniemi P, et al. Double vs single primary tooth extraction in interceptive treatment of palatally displaced canines. Angle Orthod. 2020 Nov 1;90(6):751–7. | Excluded by title/Abstarct |
|  | Shpack N, Davidovitch M, Sarne O, Panayi N, Vardimon AD. Duration and anchorage management of canine retraction with bodily versus tipping mechanics. Angle Orthod. 2008 Jan;78(1):95–100. | Excluded by title/Abstarct |
|  | Thorstenson GA, Kusy RP. Effect of archwire size and material on the resistance to sliding of self-ligating brackets with second-order angulation in the dry state. Am J Orthod Dentofacial Orthop. 2002 Sep;122(3):295–305. | Excluded by title/Abstract |
|  | Garcia-Figueroa MA, Raboud DW, Lam EW, Heo G, Major PW. Effect of buccolingual root angulation on the mesiodistal angulation shown on panoramic radiographs. Am J Orthod Dentofacial Orthop. 2008 Jul;134(1):93–9. | Excluded by title/Abstarct |
|  | Azizi F, Shahidi-Zandi V. Effect of different types of dental anchorage following first premolar extraction on mandibular third molar angulation. Int Orthod. 2018 Mar;16(1):82–90. | Excluded; non-eligible |
|  | Kamalakannan D, Anathanarayanan V, Padmanaban S. Effect of extraction or nonextraction orthodontic treatment modality on favorability of eruption of impacted third molars. Indian J Dent Res. 2019 May;30(3):428–36. | Excluded by title/Abstract |
|  | Sangcharearn Y, Ho C. Effect of Incisor Angulation on Overjet and Overbite in Class II Camouflage Treatment. A typodont study. Angle Orthod. 2007 Nov;77(6):1011–8. | Excluded by title/Abstarct |
|  | Di Giovanni T, Vogiatzi T, Koretsi V, Walsh T, Silikas N, Papageorgiou SN. Effect of orthodontic extraction of mandibular premolars on third molar angulation after treatment with fixed appliances : A cross-sectional study. J Orofac Orthop. 2024 Nov;85(6):392–403. | Excluded; non-eligible |
|  | Abohabib A, Viñas MJ, Ustrell JM. Effect of orthodontic premolar extraction on maxillary teeth angulation and arch dimensions in adolescent patients: A 3-D digital model analysis. J Clin Exp Dent. 2024 Feb;16(2):e137–44. | Excluded by title/Abstarct |
|  | Mendoza-Garc\’\ia LV, Vaillard-Jiménez E, Garc\’\ia-Rocha A, Bellot-Arc\’\is C, Paredes-Gallardo V. Effect of orthodontic treatment involving first premolar extractions on mandibular third molar angulation and retromolar space. J Clin Exp Dent. 2017 Mar;9(3):e333-e337-e333–7. | Excluded: no SD |
|  | You TM, Ban BH, Jeong JS, Huh J, Doh RM, Park W. Effect of premolar extraction and presence of the lower third molar on lower second molar angulation in orthodontic treatment. Oral Surg Oral Med Oral Pathol Oral Radiol. 2014 Sep;118(3):278–83. | Excluded: not in English |
|  | Fan L, Kuang Q, Tang Y, Qin P. [Effect of premolar extractions on third molar angulation changes: a meta-analysis]. Zhong Nan Da Xue Xue Bao Yi Xue Ban. 2015 Mar;40(3):317–25. | Excluded by title/Abstarct |
|  | Shabat MA, Bede SY. Effect of the local application of bupivacaine in early pain control following impacted mandibular third molar surgery: A randomized controlled study. Dental and Medical Problems. 2021;58(4):483–8. | Excluded by title/Abstarct |
|  | Chu Y, Zhang L, Zhao Y, Yi F, Lu Y. Effectiveness of modifications to preadjusted appliance prescriptions based on racial dental characteristics assessed by the ABO Cast-Radiograph Evaluation: A propensity score matching study. PeerJ [Internet]. 2021;9. | Excluded by title/Abstract |
|  | Guo XH, Qian YF, Feng QP. Effects of different premolar extraction on lower third molar eruption. Shanghai Kou Qiang Yi Xue. 2007 Aug;16(4):370–3. | Excluded: not in English |
|  | Bayram M, Ozer M, Arici S. Effects of first molar extraction on third molar angulation and eruption space. Oral Surg Oral Med Oral Pathol Oral Radiol Endod. 2009 Feb;107(2):e14-20-e14-20. | Excluded: no Angles |
|  | Gohilot A, Pradhan T, Keluskar KM. Effects of first premolar extraction on maxillary and mandibular third molar angulation after orthodontic therapy. J Oral Biol Craniofac Res. 2012 May;2(2):97–104. | Excluded: No ex-group |
|  | Ke HF, Wang CL, Liu L, Wei FL, Guo XX. Effects of first premolar extractions on third molar angulations. Hua Xi Kou Qiang Yi Xue Za Zhi. 2006 Jun;24(3):228–30. | Excluded by title/Abstract |
|  | He YH, Duan YZ, Pan JJ, Xi LL. Effects of the first premolar extraction on the third molar angulation. Hua Xi Kou Qiang Yi Xue Za Zhi. 2008 Aug;26(4):396–8. | Excluded: not in English |
|  | Alessandri Bonetti G, Incerti Parenti S, Ippolito DR, Gatto MR, Luigi C. Effects of ultrasonic instrumentation with different scaler-tip angulations on the shear bond strength and bond failure mode of metallic orthodontic brackets. Korean J Orthod. 2014 Jan;44(1):44–9. | Excluded by title/Abstarct |
|  | Chen Y, Hagg U, McGrath C. Elastodontic appliance assisted fixed appliance treatment: A new two-phase protocol for extraction cases. J Orthod. 2019 Sep;46(3):251–8. | Excluded by title/Abstarct |
|  | Erratum: Comparison between the designed and achieved mesiodistal angulation of maxillary canines and posterior teeth and influencing factors: First premolar extraction treatment with clear aligners (American Journal of Orthodontics & Dentofacial Orthopedics (2022) 162(2) (e63–e70), (S0889540622003043), (10.1016/j.ajodo.2022.05.006)). American Journal of Orthodontics and Dentofacial Orthopedics. 2023;164(4):464. | Excluded by title/Abstarct |
|  | Orton-Gibbs S, Crow V, Orton HS. Eruption of third permanent molars after the extraction of second permanent molars. Part 1: Assessment of third molar position and size. Am J Orthod Dentofacial Orthop. 2001 Mar;119(3):226–38. | Excluded by title/Abstract |
|  | Langer LJ, Pandis N, de la Rosa Maria R and Jost-Brinkmann PG, Bartzela TN. Eruption pattern of third molars in orthodontic patients treated with first permanent molar extraction: A longitudinal retrospective evaluation. J Clin Med. 2023 Jan;12(3):1060–1060. | Included for outcome assessment |
|  | Hsiao CC, Boynton JR. Etiology, Classification and Management of Ectopic Eruption of Permanent First Molars. J Mich Dent Assoc. 2016 Jan;98(1):26–30. | Excluded by title/Abstarct |
|  | Omidkhoda M, Radvar M, Azizi M, Dehghani M. Evaluating the Efficacy of a Modified Piezo-Puncture Method on the Rate of Tooth Movement in Orthodontic Patients: A Clinical Study. Turk J Orthod. 2020 Mar;33(1):13–20. | Excluded by title/Abstarct |
|  | Taneja IZ, Zhai G, Kravitz ND, Dischinger B, Johnston M, Kau CH, et al. Evaluating the efficiency of mandibular molar protraction using Herbst appliances versus temporary anchorage devices: a retrospective case-controlled study. Prog Orthod. 2024 Sep 2;25(1):32. | Excluded by title/Abstarct |
|  | Hoybjerg AJ, Currier GF, Kadioglu O. Evaluation of 3 retention protocols using the American Board of Orthodontics cast and radiograph evaluation. Am J Orthod Dentofacial Orthop. 2013 Jul;144(1):16–22. | Excluded by title/Abstarct |
|  | Kamisetty SK, N R, N R, N C, Dwaragesh, Praven. Evaluation of Effects and Effectiveness of Various α and β Angulations for Three Different Loop Made of Stainless Steel Arch Wires - A FEM Study. J Clin Diagn Res. 2014 Jul;8(7):ZC33-37. | Excluded by title/Abstarct |
|  | Shinde S. Evaluation of Effects of Various Alpha and Beta Angulations for T-loop Made of Stainless Steel and Titanium Molybdenum Archwires during Space Closure: Finite Element Method (FEM) Study. Mymensingh Med J. 2023 Jul;32(3):855–61. | Excluded by title/Abstarct |
|  | Park JH, Chae JM, Bay RC, Kim MJ, Lee KY, Chang NY. Evaluation of factors influencing the success rate of orthodontic microimplants using panoramic radiographs. Korean J Orthod. 2018 Jan;48(1):30–8. | Excluded by title/Abstarct |
|  | Aboalnaga AA, Fouda AS. Evaluation of the effect of extraction in comparison to distalization on the maxillary third molars in class II malocclusion: a retrospective study. Clin Oral Investig. 2024 Mar;28(3):191–191. | Excluded; non-eligible |
|  | Khouri C, Aoun G, Khouri C, Saade M, Salameh Z, Berberi A. Evaluation of Third Molar Impaction Distribution and Patterns in a Sample of Lebanese Population. J Maxillofac Oral Surg. 2022 Jun;21(2):599–607. | Excluded by title/Abstract |
|  | Edelmann AR, Hosseini B, Byrd WC, Preisser JS, Tyndall DA, Nguyen T, et al. Exploring Effectiveness of Computer-Aided Planning in Implant Positioning for a Single Immediate Implant Placement. J Oral Implantol. 2016 Jun;42(3):233–9. | Excluded by title/Abstract |
|  | Kato C, Watari I, Aida J, Ono T. Factors associated with the eruption of the impacted maxillary third molars after second molar extraction. Am J Orthod Dentofacial Orthop. 2022 Nov;162(5):636-644.e4-636-644.e4. | Excluded by title/Abstract |
|  | Hoang E, Sheller B, Greenlee G, Susarla S, Mancl L. Factors Contributing to Canine Impaction in Patients With Unilateral Cleft Lip and Palate Undergoing Alveolar Bone Grafts. J Oral Maxillofac Surg. 2023 Oct;81(10):1286–94. | Excluded by title/Abstract |
|  | Nahajowski M, Hnitecka S, Antoszewska-Smith J, Rumin K, Dubowik M, Sarul M. Factors influencing an eruption of teeth associated with a dentigerous cyst: a systematic review and meta-analysis. BMC Oral Health. 2021 Apr 7;21(1):180. | Excluded by title/Abstract |
|  | Li XT, Zhang DM, Yang YQ. Factors influencing the changes of lower third molars’ angulation and space/crown width ratio by orthodontic treatment. Beijing Da Xue Xue Bao. 2011 Apr;43(2):267–71. | Excluded: not in English |
|  | Su H, Han B, Li S, Na B, Ma W, Xu TM. Factors predisposing to maxillary anchorage loss: a retrospective study of 1403 cases. PLoS One. 2014;9(10):e109561. | Excluded by title/Abstract |
|  | Caiazzo A, Brugnami F. From Corticotomy to Orthodontically Driven Osteogenesis: Surgical Considerations. In: Orthodontically Driven Osteogenesis, Second Edition [Internet]. 2024. p. 87–110. | Excluded by title/Abstract |
|  | ElNaghy R, Daabous AT, Hasanin M, Tanha N, Ancona N, Al-Qawasmi R. Genetics of three-dimensional tooth inclination and angulation in orthodontic patients with Class I Occlusion: A cross-sectional study. Int Orthod. 2023 Mar;21(1):100713. | Excluded by title/Abstract |
|  | Santana LG, Pereira TS, Fernandes IB, Souza Margareth Maria Gomes de and Paiva SM, Ramos-Jorge ML, Marques LS. Impaction of mandibular third molars after orthodontic treatment by the edgewise method: a retrospective study. Braz Oral Res. 2020 Jun;34:e065–e065. | Excluded by title/Abstract |
|  | Phan AH, Lam PH, Le LD, Le SH. Improvement of the Impacted Level of Lower Third Molars After Orthodontic Treatment. Int Dent J. 2023 Oct;73(5):692–700. | Excluded; non-eligible |
|  | Gegler A, Fontanella V. In vitro evaluation of a method for obtaining periapical radiographs for diagnosis of external apical root resorption. Eur J Orthod. 2008 Jun;30(3):315–9. | Excluded by title/Abstract |
|  | Oduncuoğlu BF, Yamanel K, Koçak ZŞ. In Vitro Evaluation of Direct and Indirect Effects of Sonic and Ultrasonic Instrumentations on the Shear Bond Strength of Orthodontic Brackets. Turk J Orthod. 2020 Mar;33(1):37–42. | Excluded by title/Abstract |
|  | Tadic N, Woods MG. Incisal and soft tissue effects of maxillary premolar extraction in class II treatment. Angle Orthod. 2007 Sep;77(5):808–16. | Excluded by title/Abstract |
|  | Shalish M, Peck S, Wasserstein A, Peck L. Increased occurrence of dental anomalies associated with infraocclusion of deciduous molars. Angle Orthod. 2010 Dec;80(3):440–5. | Excluded by title/Abstract |
|  | Tarazona B, Paredes V, Llamas Jose-Maria and Cibrian R, Gand\’\ia JL. Influence of first and second premolar extraction or non-extraction treatments on mandibular third molar angulation and position. A comparative study. Med Oral Patol Oral Cir Bucal. 2010 Sep;15(5):e760-6-e760-6. | Included for outcome assessment |
|  | Jain S, Valiathan A. Influence of first premolar extraction on mandibular third molar angulation. Angle Orthod. 2009 Nov;79(6):1143–8. | Included for outcome assessment |
|  | Brezulier D, Fau V, Sorel O. Influence of orthodontic premolar extraction therapy on the eruption of the third molars: A systematic review of the literature. J Am Dent Assoc. 2017 Dec;148(12):903–12. | Excluded: Systematic review |
|  | Elsey MJ, Rock WP. Influence of orthodontic treatment on development of third molars. Br J Oral Maxillofac Surg. 2000 Aug;38(4):350–3. | Excluded: No Ex-Group |
|  | Al Kuwari HM, Talakey AA, Al-Sahli RM, Albadr AH. Influence of orthodontic treatment with first premolar extraction on the angulation of the mandibular third molar. Saudi Med J. 2013 Jun;34(6):639–43. | Included for outcome assessment |
|  | Pan F, Yang Z, Wang J, Cai R, Liu J, Zhang C, et al. Influence of orthodontic treatment with premolar extraction on the spatial position of maxillary third molars in adult patients: a retrospective cohort cone-bean computed tomography study. BMC Oral Health. 2020 Nov 11;20(1):321. | Excluded: no panoramic |
|  | Janson G, Branco NC, Fernandes TMF, Sathler R, Garib D, Lauris JRP. Influence of orthodontic treatment, midline position, buccal corridor and smile arc on smile attractiveness. Angle Orthod. 2011 Jan;81(1):153–61. | Excluded by title/Abstract |
|  | Durgesh BH, Gowda KHK, AlShahrani OA, Almalki AD, Almalki WD, Balharith MMS, et al. Influence of premolar extraction or non-extraction orthodontic therapy on the angular changes of mandibular third molars. Saudi J Biol Sci. 2016 Nov;23(6):736–40. | Included for outcome assessment |
|  | Feng GY, Zou BS, Gao XM, Zeng XL, Wang XJ, Yan Y. Influence of the mandibular second premolar extraction on the angulation of the mandibular third molar. Beijing Da Xue Xue Bao. 2013 Feb;45(1):77–80. | Excluded by title/Abstract |
|  | Geng J, Gu Y. Influence of the maxillary sinus floor on the orthodontic movement of maxillary first molars: a cone beam computed tomography study. Chinese Journal of Orthodontics. 2022;29(1):2–7. | Excluded by title/Abstract |
|  | Canuto LFG, de Freitas KMS, de Freitas MR, Cançado RH. Influence of treatment including second molars on final and postretention molar angulation. Dental Press J Orthod. 2013 Oct;18(5):121–7. | Excluded by title/Abstract |
|  | Livas C, Pandis N, Booij JW, Halazonetis DJ, Katsaros C, Ren Y. Influence of unilateral maxillary first molar extraction treatment on second and third molar inclination in Class II subdivision patients. Angle Orthod. 2016 Jan;86(1):94–100. | Excluded: comparison left and right side |
|  | Leethanakul C, Kanokkulchai S, Pongpanich S, Leepong N, Charoemratrote C. Interseptal bone reduction on the rate of maxillary canine retraction. Angle Orthod. 2014 Sep;84(5):839–45. | Excluded by title/Abstract |
|  | Uslu-Akçam O, Gökalp H. Investigation of effects of class II malocclusion therapy with four premolars extractions on third molar eruption. Journal of Orofacial Sciences. 2015;7(2):113–8. | Excluded: no panoramic |
|  | Moseling KP, Woods MG. Lip curve changes in females with premolar extraction or nonextraction treatment. Angle Orthod. 2004 Feb;74(1):51–62. | Excluded by title/Abstract |
|  | Stanaitytė R, Trakinienė G, Gervickas A. Lower dental arch changes after bilateral third molar removal. Stomatologija. 2014;16(1):31–6. | Excluded by title/Abstract |
|  | Teo TKY, Ashley PF, Derrick D. Lower first permanent molars: developing better predictors of spontaneous space closure. Eur J Orthod. 2016 Feb;38(1):90–5. | Excluded by title/Abstract |
|  | Xue JJ, Ye NS, Li JY, Lai WL. Management of an impacted maxillary central incisor with dilacerated root. Saudi Med J. 2013 Oct;34(10):1073–9. | Excluded by title/Abstract |
|  | Kumar M, Goyal M, Kumar S, Nongthombam H. Management of anchor loss with the use of infrazygomatic implants – A case series. Journal of Contemporary Orthodontics. 2024;8(1):76–84. | Excluded by title/Abstract |
|  | Ahmed I, Gul-e-Erum, Kumar N. Mandibular third molar angulation in extraction and non extraction orthodontic cases. J Ayub Med Coll Abbottabad. 2011 Jul;23(3):32–5. | Excluded: No SD |
|  | Koga Y, Park JH, Tai K. Maxillary canine substitution for the severely resorbed root of central incisor: 12-year follow-up. Int J Orthod Milwaukee. 2013 Winter;24(4):9–14. | Excluded by title/Abstract |
|  | Janson G, Putrick LM, Henriques JFC, de Freitas MR, Henriques RP. Maxillary third molar position in Class II malocclusions: the effect of treatment with and without maxillary premolar extractions. Eur J Orthod. 2006 Dec;28(6):573–9. | Excluded: No comparison post- and pre-treatment |
|  | Putri AM, Sumardi S, Soegiharto BM. Mesioangular impacted lower third molar angulation changes in non-extraction orthodontic treatment. In 2018. | Excluded; non-eligible |
|  | Hartono DF, Soegiharto BM, Sumardi S. Mesioangular impacted lower third molars: Angulation before and after orthodontic treatment with premolar extraction. In 2018. | Excluded: no ex-group |
|  | Ueda H, Masunaga M, Horie K, Medina CC, Tanne K. Mesiodistal angulation of the lateral teeth to the functional occlusal plane in normal occlusions. APOS Trends in Orthodontics. 2016;6(6):302–5. | Excluded by title/Abstract |
|  | Heckmann K, Spitzer WJ, Lisson JA. Molar inclination in panoramic x-rays as an indicator for extraction decisions. J Orofac Orthop. 2007 Nov;68(6):491–500. | Excluded by title/Abstract |
|  | Nallanchakrava S, Mettu S, Reddy NG, Jangam K. Multidisciplinary Approach for the Management of Dilacerated Permanent Maxillary Incisor: A Case Report. Int J Clin Pediatr Dent. 2020 Dec;13(6):725–8. | Excluded by title/Abstract |
|  | Islam MM, Hossain MZ, Rahman MM. Orthodontic management of a young girl with class I malocclusion and severe crowding: a case report. Mymensingh Med J. 2015 Apr;24(2):399–403. | Excluded by title/Abstract |
|  | Islam MM, Hossain MZ. Orthodontic Management of A Young Girl with Class II Div1 Malocclusion. Mymensingh Med J. 2015 Oct;24(4):845–50. | Excluded by title/Abstract |
|  | Farronato G, Maspero C, Farronato D. Orthodontic movement of a dilacerated maxillary incisor in mixed dentition treatment. Dent Traumatol. 2009 Aug;25(4):451–6. | Excluded by title/Abstract |
|  | Delsol L, Bousquet P. [Orthodontic treatment of gingival recession: indications]. Orthod Fr. 2011 Sep;82(3):269–78. | Excluded by title/Abstract |
|  | Deguchi T, Terao F, Aonuma T, Kataoka T, Sugawara Y, Yamashiro T, et al. Outcome assessment of lingual and labial appliances compared with cephalometric analysis, peer assessment rating, and objective grading system in Angle Class II extraction cases. Angle Orthod. 2015 Dec;85(3):400–7. | Excluded by title/Abstract |
|  | Akinci Cansunar H, Uysal T. Outcomes of different Class II treatments : Comparisons using the American Board of Orthodontics Model Grading System. J Orofac Orthop. 2016 Jul;77(4):233–41. | Excluded by title/Abstract |
|  | D’Agostini Derech C, Locks A, Bolognese AM. Palatal configuration in Class II Division 1 malocclusion: A longitudinal study. American Journal of Orthodontics and Dentofacial Orthopedics. 2010;137(5):658–64. | Excluded by title/Abstract |
|  | Niedzielska IA, Drugacz J, Kus N, Kreska J. Panoramic radiographic predictors of mandibular third molar eruption. Oral Surg Oral Med Oral Pathol Oral Radiol Endod. 2006 Aug;102(2):154–8; discussion 159-154–8; discussion 159. | Excluded by title/Abstract |
|  | Chen H, Han B, Jiang R, Su H, Feng T, Teng F, et al. PASS versus MBTTM for evaluation of anchorage control in three-dimensional measurements: a randomized controlled trial. Eur J Orthod. 2021 Jan 29;43(1):113–9. | Excluded by title/Abstract |
|  | Güngörmüs M. Pathologic status and changes in mandibular third molar position during orthodontic treatment. J Contemp Dent Pract. 2002 May;3(2):11–22. | Excluded; non-eligible |
|  | Promchaiwattana P, Suzuki B, Krisanaprakornkit S, Suzuki EY. Periodontal ligament enhancement in mesio-angulated impaction of third molars after orthodontic tooth movement: A prospective cohort study. Am J Orthod Dentofacial Orthop. 2020 Oct;158(4):495–504. | Excluded by title/Abstract |
|  | Montenegro VCJ, Jones A, Petocz P, Gonzales C, Darendeliler MA. Physical properties of root cementum: Part 22. Root resorption after the application of light and heavy extrusive orthodontic forces: a microcomputed tomography study. Am J Orthod Dentofacial Orthop. 2012 Jan;141(1):e1-9. | Excluded by title/Abstract |
|  | Volchansky A, Evans B, Cleaton-Jones P. Position and orientation of the maxillary first permanent molar and localized attachment loss. World J Orthod. 2004 Summer;5(2):141–6. | Excluded by title/Abstract |
|  | Bolooki H, Hameed O, Sherriff M, Minhas G. Positional factors affecting the surgical management of impacted permanent mandibular canines. J Orthod. 2022 Dec;49(4):441–7. | Excluded by title/Abstract |
|  | Tepedino M, Franchi L, Fabbro O, Chimenti C. Post-orthodontic lower incisor inclination and gingival recession-a systematic review. Prog Orthod. 2018 Jun 18;19(1):17. | Excluded by title/Abstract |
|  | Janson G, Rizzo M, Laranjeira V, Garib DG, Valarelli FP. Posterior teeth angulation in non-extraction and extraction treatment of anterior open-bite patients. Prog Orthod. 2017 Dec;18(1):13. | Excluded by title/Abstract |
|  | Wes Fleming J, Buschang PH, Kim KB, Oliver DR. Posttreatment occlusal variability among angle Class I nonextraction patients. Angle Orthod. 2008 Jul;78(4):625–30. | Excluded by title/Abstract |
|  | Moreira FC, Vaz LG, Guastaldi AC, English JD, Jacob HB. Potentialities and limitations of computer-aided design and manufacturing technology in the nonextraction treatment of Class I malocclusion. Am J Orthod Dentofacial Orthop. 2021 Jan;159(1):86–96. | Excluded by title/Abstract |
|  | Behbehani F, Artun J, Thalib L. Prediction of mandibular third-molar impaction in adolescent orthodontic patients. Am J Orthod Dentofacial Orthop. 2006 Jul;130(1):47–55. | Excluded by title/Abstract |
|  | Warford JHJ, Grandhi RK, Tira DE. Prediction of maxillary canine impaction using sectors and angular measurement. Am J Orthod Dentofacial Orthop. 2003 Dec;124(6):651–5. | Excluded by title/Abstract |
|  | Artun J, Behbehani F, Thalib L. Prediction of maxillary third molar impaction in adolescent orthodontic patients. Angle Orthod. 2005 Nov;75(6):904–11. | Excluded by title/Abstract |
|  | Smith B, Stewart K, Liu S, Eckert G, Kula K. Prediction of orthodontic treatment of surgically exposed unilateral maxillary impacted canine patients. Angle Orthod. 2012 Jul;82(4):723–31. | Excluded by title/Abstract |
|  | Shukla NK, Sharma VK, Chaturvedi TP, Parihar AV, Chugh VK. Prediction of spontaneous eruption of palatally displaced canine based on variables measurements in panoramic radiograph: A systematic review. J Oral Biol Craniofac Res. 2021 Jun;11(2):344–53. | Excluded: systematic review |
|  | Brignardello-Petersen R. Premolar extraction as part of orthodontic treatment may result in a small increase in retromolar space and improve third-molar angulation. J Am Dent Assoc. 2017 Aug;148(8):e119. | Excluded; non-eligible |
|  | Stivaros N, Mandall NA. Radiographic factors affecting the management of impacted upper permanent canines. J Orthod. 2000 Jun;27(2):169–73. | Excluded by title/Abstract |
|  | Patel S, Ashley P, Noar J. Radiographic prognostic factors determining spontaneous space closure after loss of the permanent first molar. Am J Orthod Dentofacial Orthop. 2017 Apr;151(4):718–26. | Excluded by title/Abstract |
|  | Upadhyay M. Re: The effects of first premolar extractions on third molar angulations. Angle Orthod. 2006 Nov;76(6):iii; author reply iii. | Excluded by title/Abstract |
|  | Azizi F, Zadeh AN, Golshah A, Imani MM, Safari-Faramani R. Relationship between the amount of mesial movement of the mandibular first molar and angular changes of the mandibular third molar: A retrospective comparative study in growing patients with and without first premolar extraction. Int Orthod. 2023 Sep;21(3):100779–100779. | Excluded; non-eligible |
|  | Smailienė D, Trakinienė G, Beinorienė A, Tutlienė U. Relationship between the Position of Impacted Third Molars and External Root Resorption of Adjacent Second Molars: A Retrospective CBCT Study. Medicina (Kaunas). 2019 Jun 24;55(6). | Excluded by title/Abstract |
|  | Tang Z, Chen W, Mei L, Abdulghani EA, Zhao Z, Li Y. Relative anchorage loss under reciprocal anchorage in mandibular premolar extraction cases treated with clear aligners. Angle Orthod. 2023 Jul 1;93(4):375–81. | Excluded by title/Abstract |
|  | Gupta DK, Aggarwal K. Root-crown ratio: a new method for measurement of orthodontic external apical root resorption. Orthodontic Waves. 2020;79(1):23–9. | Excluded by title/Abstract |
|  | Patrick T, Stickrath K, Christensen J, Jacox L, Mitchell K. Second molar eruption disturbances in borderline extraction orthodontic patients. Am J Orthod Dentofacial Orthop. 2024 Aug; | Excluded by title/Abstract |
|  | Cavalcante RC, Corso PFCDL, Trento GDS, Antonini F, Rebellato NLB, Costa DJD, et al. Segmental orthognathic surgery for Bolton discrepancy correction. Oral and Maxillofacial Surgery Cases. 2018;4(3):108–14. | Excluded by title/Abstract |
|  | Arrubla-Escobar D, Barbosa-Liz DM, Zapata-Noreña O, Carvajal-Flórez A, Correa-Mullet K, Gómez-Gómez SL, et al. Smile Aesthetics Assessment in Patients Undergoing the Finishing Phase of Orthodontic Treatment: An Observational Cross-Sectional Study. Cureus. 2023 Sep;15(9):e45644. | Excluded by title/Abstract |
|  | Gong Y, Li P lun, Wang H hong, Yu Q, Wei B, Shen G. [Soft tissue angle evaluation of fixed Twin-Block appliance treatment and tooth extraction treatment in skeletal Class II malocclusion]. Shanghai Kou Qiang Yi Xue. 2016 Feb;25(1):82–6. | Excluded by title/Abstract |
|  | Kundra R, Sharma D, Kaur R, Monga S, Duvedi S. Spatial and angular alterations in the dental arch after premature loss of primary molars: Immediate and 9-month computed tomography evaluation. J Indian Soc Pedod Prev Dent. 2024 Jul 1;42(3):226–34. | Excluded by title/Abstract |
|  | Ciftci V, Guney AU, Deveci C, Sanri IY, Salimow F, Tuncer AH. Spontaneous space closure following the extraction of the first permanent mandibular molar. Niger J Clin Pract. 2021 Oct;24(10):1450–6. | Excluded by title/Abstract |
|  | Garib DG, Bressane LB, Janson G, Gribel BF. Stability of extraction space closure. Am J Orthod Dentofacial Orthop. 2016 Jan;149(1):24–30. | Excluded by title/Abstract |
|  | Carruitero MJ, Aliaga-Del Castillo A, Garib D, Janson G. Stability of maxillary interincisor diastema closure after extraction orthodontic treatment. Angle Orthod. 2020 Sep 1;90(5):627–33. | Excluded by title/Abstract |
|  | Gopal H, Das SK, Barik AK, Mishra M, Rath SK, Samal R, et al. Success rate of infrazygomatic crest mini-implants used for en-masse retraction of maxillary anterior teeth in first premolar extraction cases: A three-dimensional comparative prospective clinical trial between adolescents and young adults. J World Fed Orthod. 2023 Oct;12(5):197–206. | Excluded by title/Abstract |
|  | La Monaca G, Vozza I, Annibali S, Giardino R, Pranno N, Cristalli MP. Surgical approach to malformation of maxillary central incisor following trauma to its predecessor. Two case reports. Ann Stomatol (Roma). 2017 Dec;8(3):131–8. | Excluded by title/Abstract |
|  | Tsai TP. Surgical repositioning of an impacted dilacerated incisor in mixed dentition. J Am Dent Assoc. 2002 Jan;133(1):61–6. | Excluded by title/Abstract |
|  | Brezniak N, Goren S, Zoizner R, Shochat T, Dinbar A, Wasserstein A, et al. The accuracy of the cementoenamel junction identification on periapical films. Angle Orthod. 2004 Aug;74(4):496–500. | Excluded by title/Abstract |
|  | Kuc AE, Kotuła J, Nawrocki J, Babczyńska A, Lis J, Kawala B, et al. The Assessment of the Rank of Torque Control during Incisor Retraction and Its Impact on the Resorption of Maxillary Central Incisor Roots According to Incisive Canal Anatomy—Systematic Review. Journal of Clinical Medicine [Internet]. 2023;12(8). Available from: https://www.scopus.com/inward/record.uri?eid=2-s2.0-85154566013&doi=10.3390%2fjcm12082774&partnerID=40&md5=268b261d1490c3d2f64780bcc2b31718 | Excluded by title/Abstract |
|  | Miclotte A, Franco A, Guerrero ME, Willems G, Jacobs R. The association between orthodontic treatment and third molar position, inferior alveolar nerve involvement, and prediction of wisdom tooth eruption. Surg Radiol Anat. 2015 Dec;37(4):333–9. | Excluded by title/Abstract |
|  | Russell B, Skvara M, Draper E, Proffit WR, Philips C, White Jr RP. The association between orthodontic treatment with removal of premolars and the angulation of developing mandibular third molars over time. Angle Orthod. 2013 May;83(3):376–80. | Included for outcome assessment |
|  | Devine CP, Patel D, Pandis N, Fleming PS. The development of a novel Orthodontic Alignment Index and its use to evaluate the effect of residual overjet on the stability of the alignment of the maxillary anterior dentition. Prog Orthod. 2022 Dec 28;23(1):56. | Excluded by title/Abstract |
|  | Miclotte A, Grommen B, de Llano-Pérula M, Verdonck A, Jacobs R, Willems G. The effect of first and second premolar extractions on third molars: A retrospective longitudinal study. J Dent. 2017 Jun;61:55–66. | Excluded; non-eligible |
|  | Xie JZ. The effect of first premolar extraction on third molar angulation and eruption space. Shanghai Kou Qiang Yi Xue. 2009 Dec;18(6):584–7. | Excluded: not in English |
|  | Miclotte A, Grommen B, Lauwereins S, Cadenas de Llano-Pérula M, Alqerban A, Verdonck A, et al. The effect of headgear on upper third molars: a retrospective longitudinal study. Eur J Orthod. 2017 Aug 1;39(4):426–32. | Excluded by title/Abstract |
|  | Ren S, Gu Y. The effect of orthodontic treatment with first premolar extraction on eruption of mandibular third molar : a cone beam CT study. Chinese Journal of Orthodontics. 2022;29(3):157–60. | Excluded: not in English |
|  | Shen G, Chen RJ, Hu Z, Qian YF. The effects of a newly designed twin-slot bracket on severely malpositioned teeth--a typodont experimental study. Eur J Orthod. 2008 Aug;30(4):401–6. | Excluded by title/Abstract |
|  | Saysel MY, Meral GD, Kocadereli I, Taşar F. The effects of first premolar extractions on third molar angulations. Angle Orthod. 2005 Sep;75(5):719–22. | Excluded; non-eligible |
|  | Türedi M, Yazıcıoğlu S. The Effects of Increased Maxillary Canine Bracket Angulation on Tooth Movement and Alignment Efficiency: A Prospective Clinical Study. Turk J Orthod. 2022 Mar;35(1):33–8. | Excluded by title/Abstract |
|  | Lee CKJ, Quah B, Ng WH, Lai CWM, Yong CW. The effects of VY closure on lip morphology after Le Fort I maxillary advancement: A systematic review. J Stomatol Oral Maxillofac Surg. 2024 Oct;125(5):101755. | Excluded by title/Abstract |
|  | Omal PM, Thomas S, John J, George B, Mathew A. The extend of root angulation in patients visiting a dental school in south kerala: A panoramic radiographic study. Journal of Indian Academy of Oral Medicine and Radiology. 2012;24(3):186–9. | Excluded by title/Abstract |
|  | Su L, Luo C, Song H, Wang YP, Ab Rahman N. The Fabrication of a Customized Surgical Template for a Miniscrew Placement Using a Fully Digitized Process. Cureus. 2024 Apr;16(4):e58119. | Excluded by title/Abstract |
|  | Bayramoglu Z, Miloglu O, Yozgat Ilbaş F. The findings of impacted and transmigrated maxillary and mandibular canines: a retrospective cone beam computed tomography study. Minerva Dent Oral Sci. 2023 Apr;72(2):90–8. | Excluded by title/Abstract |
|  | Brezniak N, Birnboim-Blau G, Bar-Hama P, Zoizner R, Dinbar A, Wasserstein A. [The inaccuracy of the panoramic radiograph as a tool to determine tooth inclination]. Refuat Hapeh Vehashinayim (1993). 2012 Jan;29(1):36–9, 65. | Excluded by title/Abstract |
|  | Yang T, Jiang L, Sun W, Zhu M, Jiang K, Li H, et al. The incidence and severity of open gingival embrasures in adults treated with clear aligners and fixed appliances: a retrospective cohort study. Head Face Med. 2023 Jul 17;19(1):30. | Excluded by title/Abstract |
|  | Cassetta M, Altieri F. The influence of mandibular third molar germectomy on the treatment time of impacted mandibular second molars using brass wire: a prospective clinical pilot study. Int J Oral Maxillofac Surg. 2017 Jul;46(7):905–11. | Excluded by title/Abstract |
|  | Turley PK. The management of mesially inclined/impacted mandibular permanent second molars. J World Fed Orthod. 2020 Oct;9(3S):S45–53. | Excluded by title/Abstract |
|  | Tilen R, Patcas R, Bornstein MM, Ludwig B, Schätzle M. The nasopalatine canal, a limiting factor for temporary anchorage devices: a cone beam computed tomography data study. Eur J Orthod. 2017 Nov 30;39(6):646–53. | Excluded by title/Abstract |
|  | Kirschen RH, O’higgins EA, Lee RT. The Royal London Space Planning: an integration of space analysis and treatment planning: Part I: Assessing the space required to meet treatment objectives. Am J Orthod Dentofacial Orthop. 2000 Oct;118(4):448–55. | Excluded by title/Abstract |
|  | Naoumova J, Kjellberg H. The use of panoramic radiographs to decide when interceptive extraction is beneficial in children with palatally displaced canines based on a randomized clinical trial. Eur J Orthod. 2018 Nov 30;40(6):565–74. | Excluded by title/Abstract |
|  | Ongkorahadjo A, Kusnoto B. The use of pre-implantation tooth lengths in the treatment of avulsed teeth. J Clin Pediatr Dent. 2000 Winter;24(2):91–5. | Excluded by title/Abstract |
|  | Blazejewski SW 3rd. Thermoplastic inclined plane aligner for correction of bilateral mandibular canine tooth distoclusion in a cat. J Vet Dent. 2013 Winter;30(4):236–47. | Excluded by title/Abstract |
|  | Gümüş EB, Esenlik E, Kayafoğlu GE, Yıldırım M. Third molar angulation and retromolar space after functional orthodontic treatment : Evaluation of panoramic radiographs after monoblock or Herbst appliance. J Orofac Orthop. 2024 Aug;85(Suppl 2):181–8. | Excluded by title/Abstract |
|  | Shashidhar K, Castelino CK, Kuttappa MN, Nair RA, Soans CR, Nair HS. Third molar angulation changes in class II div I malocclusion subjects treated with extraction of four premolars: A retrospective study. J Int Soc Prev Community Dent. 2020 Sep;10(5):591–6. | Excluded: no panoramic |
|  | Artun J, Thalib L, Little RM. Third molar angulation during and after treatment of adolescent orthodontic patients. Eur J Orthod. 2005 Dec;27(6):590–6. | Excluded: no Panoramic |
|  | Peña-Reyes D, Freitas JQ, Freitas KMS, Bellini-Pereira SA, Aliaga-Del Castillo A, Janson G, et al. Third molar comparison in Class I and II extraction and non-extraction orthodontic treatment: A retrospective longitudinal study. Turk J Orthod. 2024 Mar;37(1):7–13. | Included for outcome assessment |
|  | Butaye C, Miclotte A, Begnoni G, Zhao Z, Zong C, Willems G, et al. Third molar position after completion of orthodontic treatment: a prospective follow-up. Dentomaxillofac Radiol. 2023 Jul;52(5):20220432. | Excluded; non-eligible |
|  | Fafat KKK, Jain D, Pandit SK, Kharbanda R, Trivedy P, Gupta E. Three Dimensional Evaluation of Accuracy of Bracket Positioning. J Pharm Bioallied Sci. 2023 Jul;15(Suppl 2):S1188–91. | Excluded by title/Abstract |
|  | Cho MY, Choi JH, Lee SP, Baek SH. Three-dimensional analysis of the tooth movement and arch dimension changes in Class I malocclusions treated with first premolar extractions: a guideline for virtual treatment planning. Am J Orthod Dentofacial Orthop. 2010 Dec;138(6):747–57. | Excluded by title/Abstract |
|  | Akın Ş, Camcı H. Three-dimensional assessment of two different canine retraction techniques: a randomized split-mouth clinical trial. Prog Orthod. 2021 Aug 9;22(1):24. | Excluded by title/Abstract |
|  | Keener DJ, de Oliveira Ruellas AC, Aliaga-Del Castillo A, Arriola-Guillén LE, Bianchi J, Oh H, et al. Three-dimensional decision support system for treatment of canine impaction. Am J Orthod Dentofacial Orthop. 2023 Oct;164(4):491–504. | Excluded by title/Abstract |
|  | Huh J, Liu J, Yu JH, Choi YJ, Ahn HK, Chung CJ, et al. Three-dimensional evaluation of a virtual setup considering the roots and alveolar bone in molar distalization cases. Sci Rep. 2023 Sep 11;13(1):14955. | Excluded by title/Abstract |
|  | Khalil RA, Salem WS. Three-dimensional evaluation of the airway morphology after miniscrew-supported en masse retraction in adult bimaxillary protrusion patients by using cone beam computed tomography: A single-arm clinical trial. Int Orthod. 2024 Oct 28;23(1):100936. | Excluded by title/Abstract |
|  | Ogura H, Numazaki K, Oyanagi T, Seiryu M, Ito A, Noguchi T, et al. Three-Dimensional Evaluation of Treatment Effects and Post-Treatment Stability of Maxillary Molar Intrusion Using Temporary Anchorage Devices in Open Bite Malocclusion. Journal of Clinical Medicine [Internet]. 2024;13(10). | Excluded by title/Abstract |
|  | Haider O, Sharaf MA, Abdulqader AA, Alhashimi N, Sharhan HM, Chen C, et al. Three-dimensional relationship between the degree of bilateral impacted mandibular third molars angulation and the mandibular dental arch parameters: a cross-sectional comparative study. Clin Oral Investig. 2023 Aug;27(8):4301–11. | Excluded by title/Abstract |
|  | Kapoor V, Kumar N, Dahiya K, Sikka R, Sirana P, Singh A. To Assess and Evaluate the Variation of Mandibular Anatomy Using Cone Beam Computed Tomography Before Planning an Implant Surgery: A Population-based Retrospective Study. J Contemp Dent Pract. 2018 Nov 1;19(11):1381–5. | Excluded by title/Abstract |
|  | Lee SJ, Kim TW, Nahm DS. Transverse implications of maxillary premolar extraction in Class III presurgical orthodontic treatment. Am J Orthod Dentofacial Orthop. 2006 Jun;129(6):740–8. | Excluded by title/Abstract |
|  | Miresmaeili A, Basafa M, Mahvelati Shamsabadi R, Farhadian N, Moghymbeigi A, Mollabashi V. Treatment decision analysis for palatally-displaced canines based on orthodontists’ opinion and CBCT. Int Orthod. 2017 Dec;15(4):625–39. | Excluded by title/Abstract |
|  | Pavlidis D, Daratsianos N, Jäger A. Treatment of an impacted dilacerated maxillary central incisor. Am J Orthod Dentofacial Orthop. 2011 Mar;139(3):378–87. | Excluded by title/Abstract |
|  | Mislik B, Konstantonis D, Katsadouris A, Eliades T. University clinic and private practice treatment outcomes in Class I extraction and nonextraction patients: A comparative study with the American Board of Orthodontics Objective Grading System. Am J Orthod Dentofacial Orthop. 2016 Feb;149(2):253–8. | Excluded by title/Abstract |
|  | Sharma VK, Shukla NK, Chaturvedi TP, Singh S. Variables to predict spontaneous eruption of palatally displaced permanent canine after interceptive extraction of primary canine: A systematic review and meta-analysis. Int Orthod. 2021 Mar;19(1):25–36. | Excluded by title/Abstract |

**Table S3: Leave-one-out method (Sensitivity Analysis)**

|  | **Analysis** | **Study_Removed** | **TE** | **lower_CI** | **upper_CI** | **pval** |
| --- | --- | --- | --- | --- | --- | --- |
|  |  |  |  |  |  |  |
| Langer et al. | Mandibular (M3L/PP , M3L/MP , M3L/M2L) | Langer et al. | -0.33705958 | -0.5797310 | -0.09438814 | 0.0118890391 |
| Tarazona et al. | Mandibular (M3L/PP , M3L/MP , M3L/M2L) | Tarazona et al. | -0.40739679 | -0.6742520 | -0.14054163 | 0.0078403769 |
| Jain and Valiathan | Mandibular (M3L/PP , M3L/MP , M3L/M2L) | Jain and Valiathan | -0.32510810 | -0.5128066 | -0.13740965 | 0.0035204291 |
| Al Kuwari et al. | Mandibular (M3L/PP , M3L/MP , M3L/M2L) | Al Kuwari et al. | -0.33342481 | -0.5517619 | -0.11508775 | 0.0072224954 |
| Peña-Reyes et al. | Mandibular (M3L/PP , M3L/MP , M3L/M2L) | Peña-Reyes et al. | -0.43142667 | -0.6740484 | -0.18880489 | 0.0034353666 |
| Singh et al. | Mandibular (M3L/PP , M3L/MP , M3L/M2L) | Singh et al. | -0.39306092 | -0.6311175 | -0.15500439 | 0.0046605952 |
| Durgesh et al. | Mandibular (M3L/PP , M3L/MP , M3L/M2L) | Durgesh et al. | -0.33493884 | -0.5901470 | -0.07973068 | 0.0157308166 |
| Russell et al. | Mandibular (M3L/PP , M3L/MP , M3L/M2L) | Russell et al. | -0.39228553 | -0.6630121 | -0.12155894 | 0.0102095467 |
| de la Rosa et al. | Maxilla (M3U/PP) | de la Rosa et al. | -0.71106006 | -1.1223387 | -0.29978144 | 0.0175956538 |
| Langer et al. | Maxilla  (M3U/PP) | Langer et al. | -0.30363595 | -0.7824511 | 0.17517924 | 0.1369049524 |
| Peña-Reyes et al. | Maxilla  (M3U/PP) | Peña-Reyes et al. | -0.36226851 | -1.4414141 | 0.71687705 | 0.2854666424 |
| de la Rosa et al. | Maxilla  (M3U/IOP) | de la Rosa et al. | -0.86454128 | -1.3359132 | -0.39316932 | 0.0003246889 |
| Langer et al. | Maxilla  (M3U/IOP) | Langer et al. | -0.09853593 | -1.9620999 | 1.76502807 | 0.6233910890 |
| de la Rosa et al. | Maxilla  (M2U/IOP) | de la Rosa et al. | -0.93375206 | -1.4086206 | -0.45888350 | 0.0001162266 |
| Langer et al. | Maxilla  (M2U/IOP) | Langer et al. | -0.36000309 | -2.8633947 | 2.14338853 | 0.3187871870 |
| Singh et al. | Maxilla  (M3L/M2L) | Singh et al. | -0.35975797 | -0.8553395 | 0.13582357 | 0.0890272700 |
| Durgesh et al. | Maxilla  (M3L/M2L) | Durgesh et al. | -0.16587651 | -0.3645356 | 0.03278260 | 0.0694975221 |
| Russell et al. | Maxilla  (M3L/M2L) | Russell et al. | -0.36835949 | -2.7726523 | 2.03593335 | 0.3021003802 |

**Figure S1:**

**
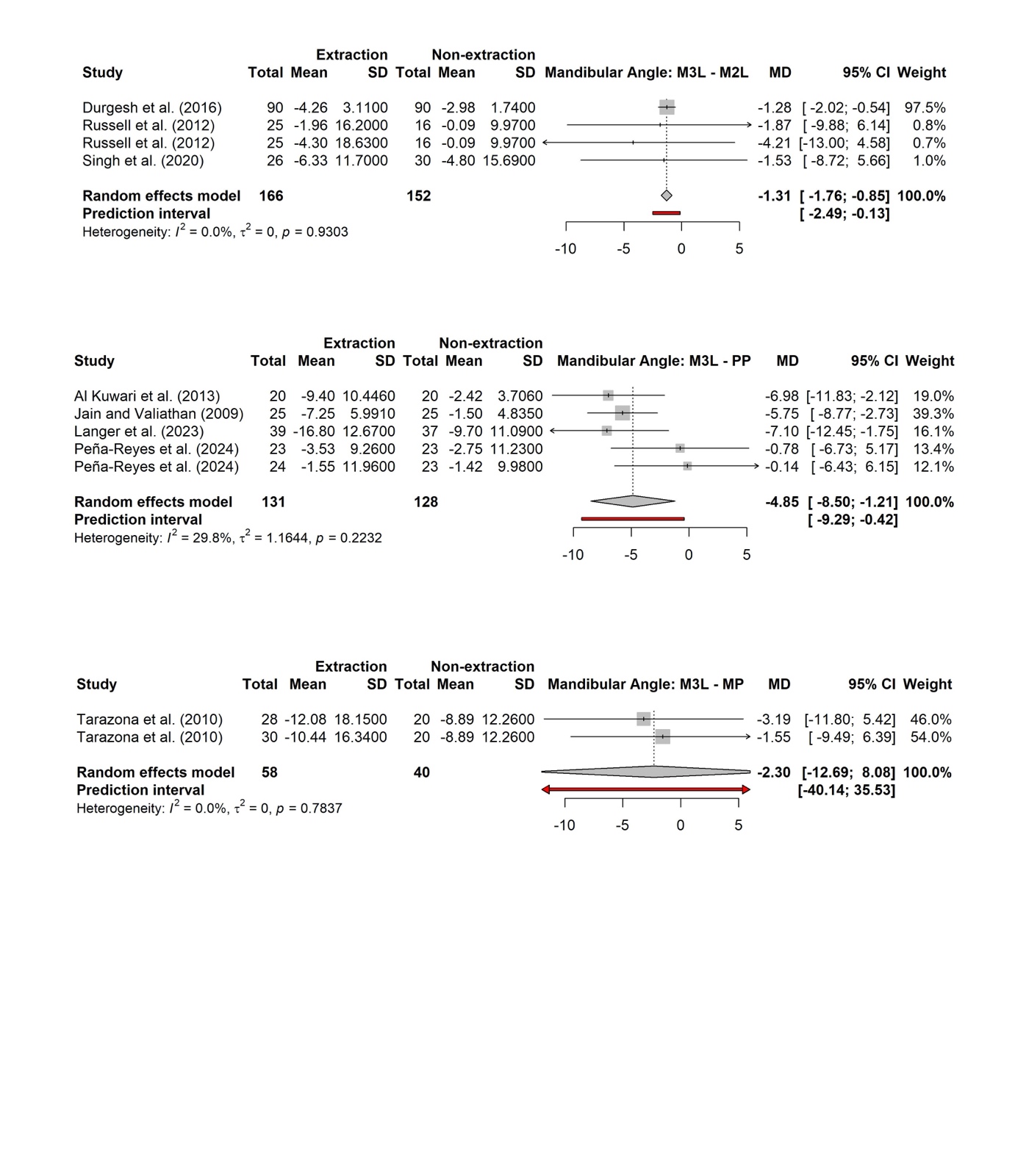
**

**Figure S2:**

**
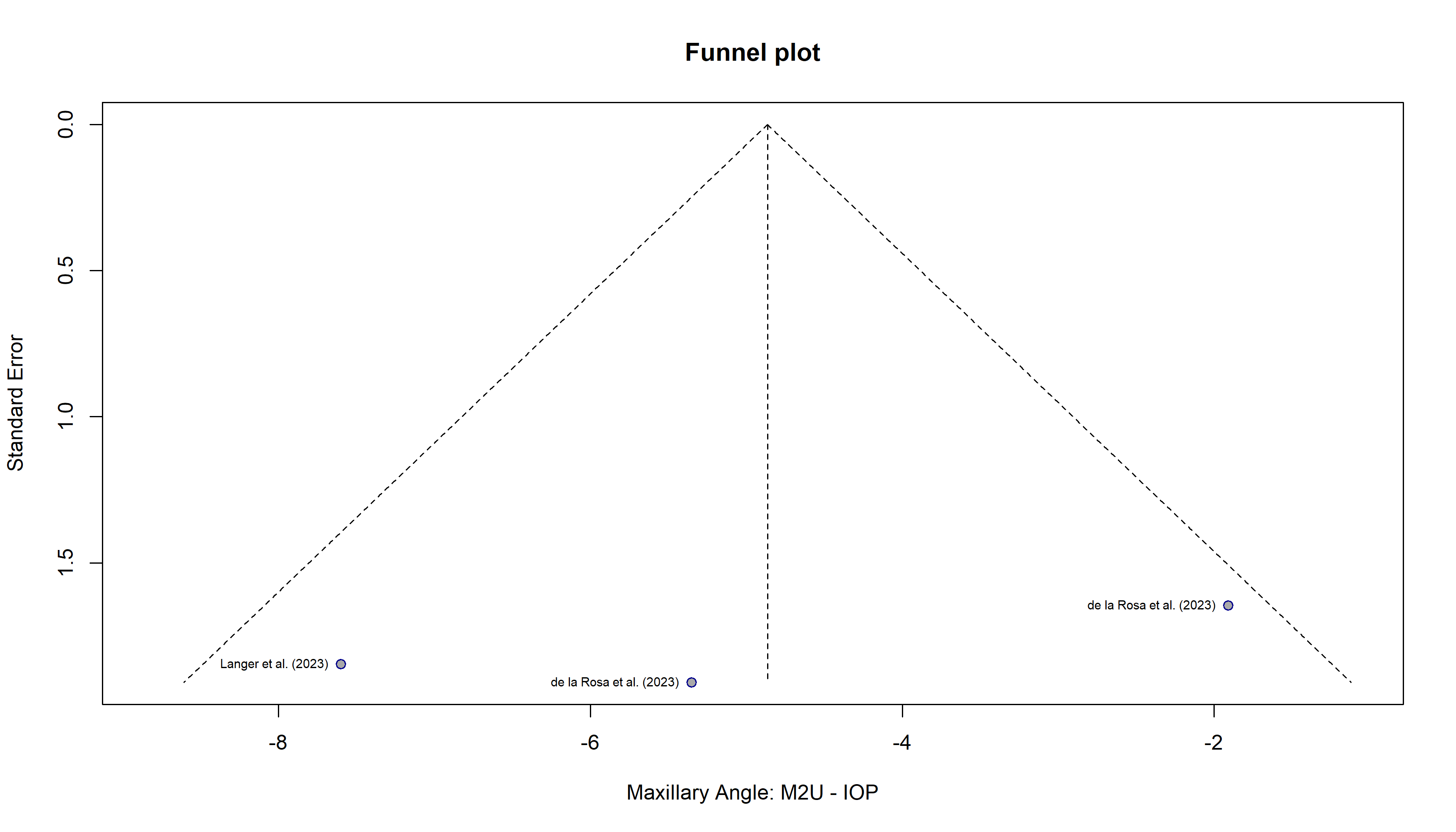

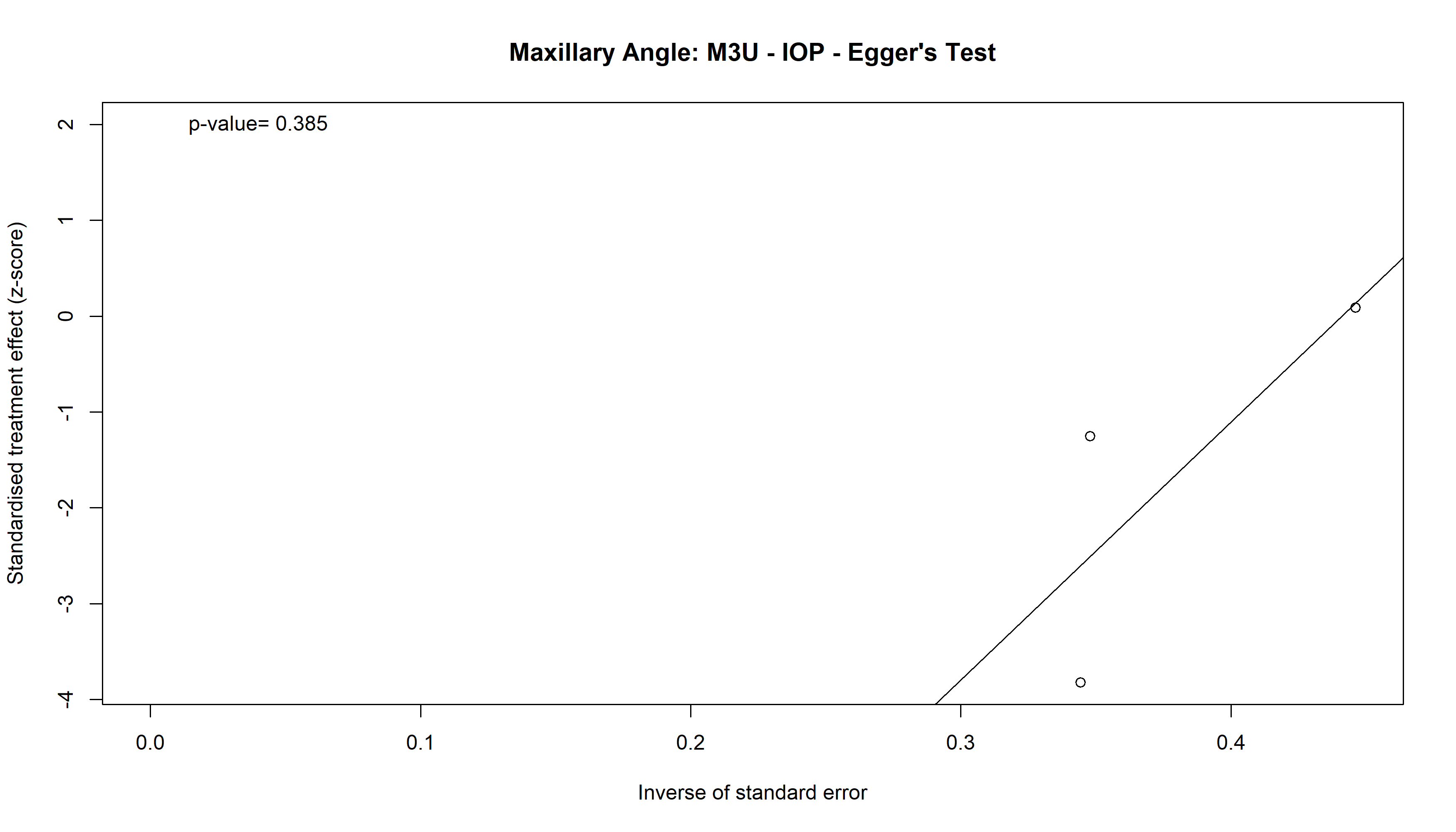

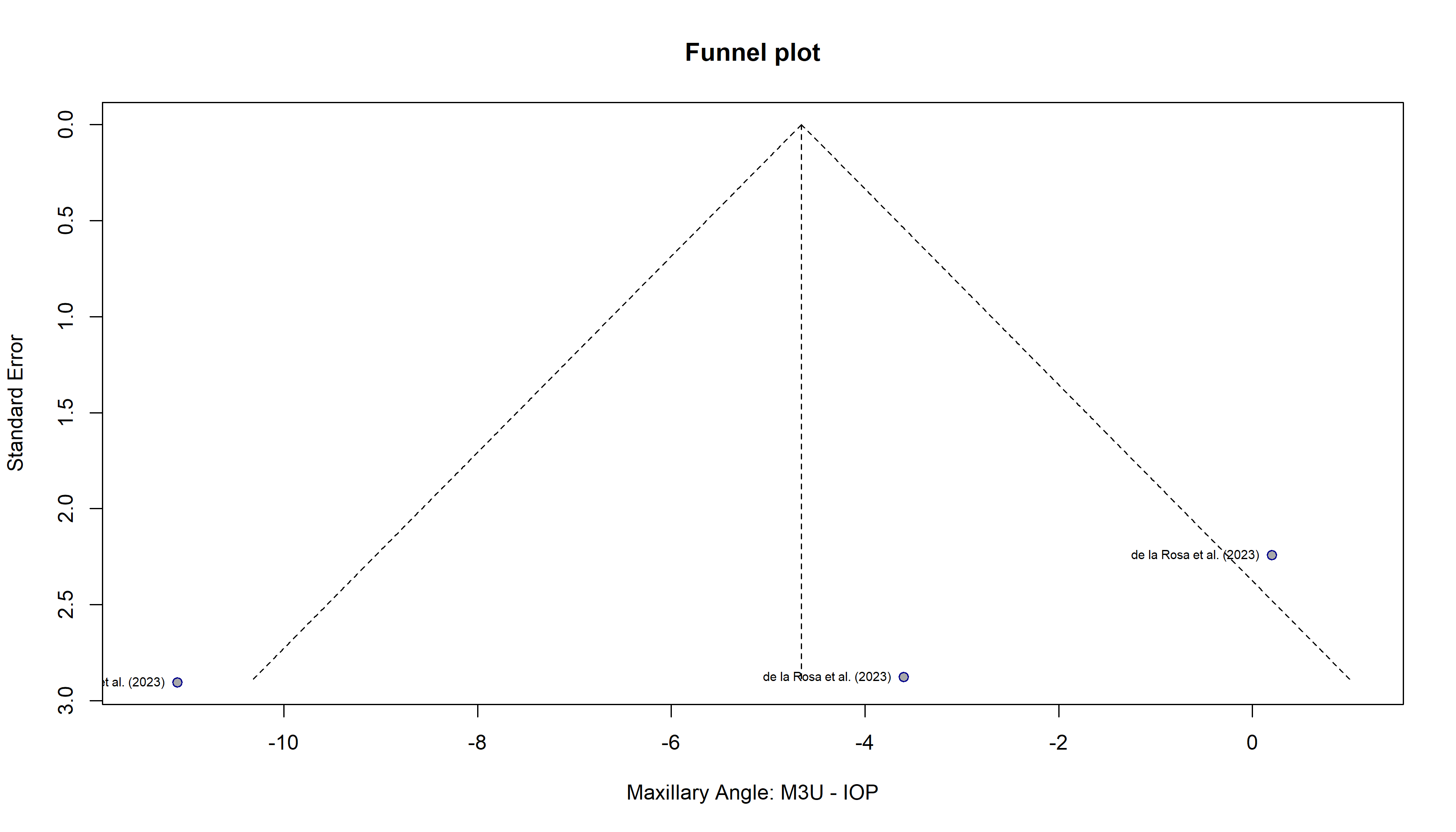

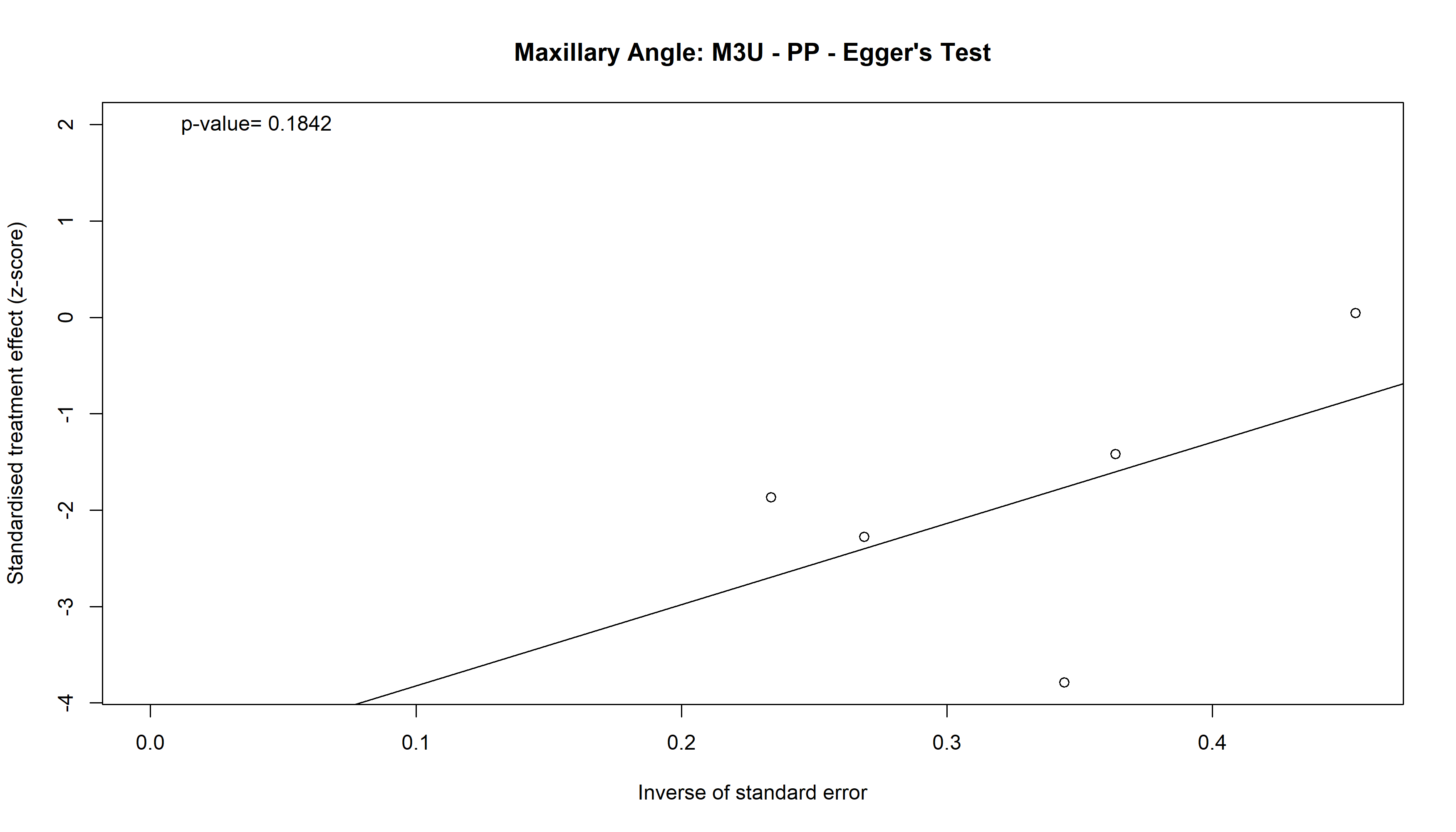

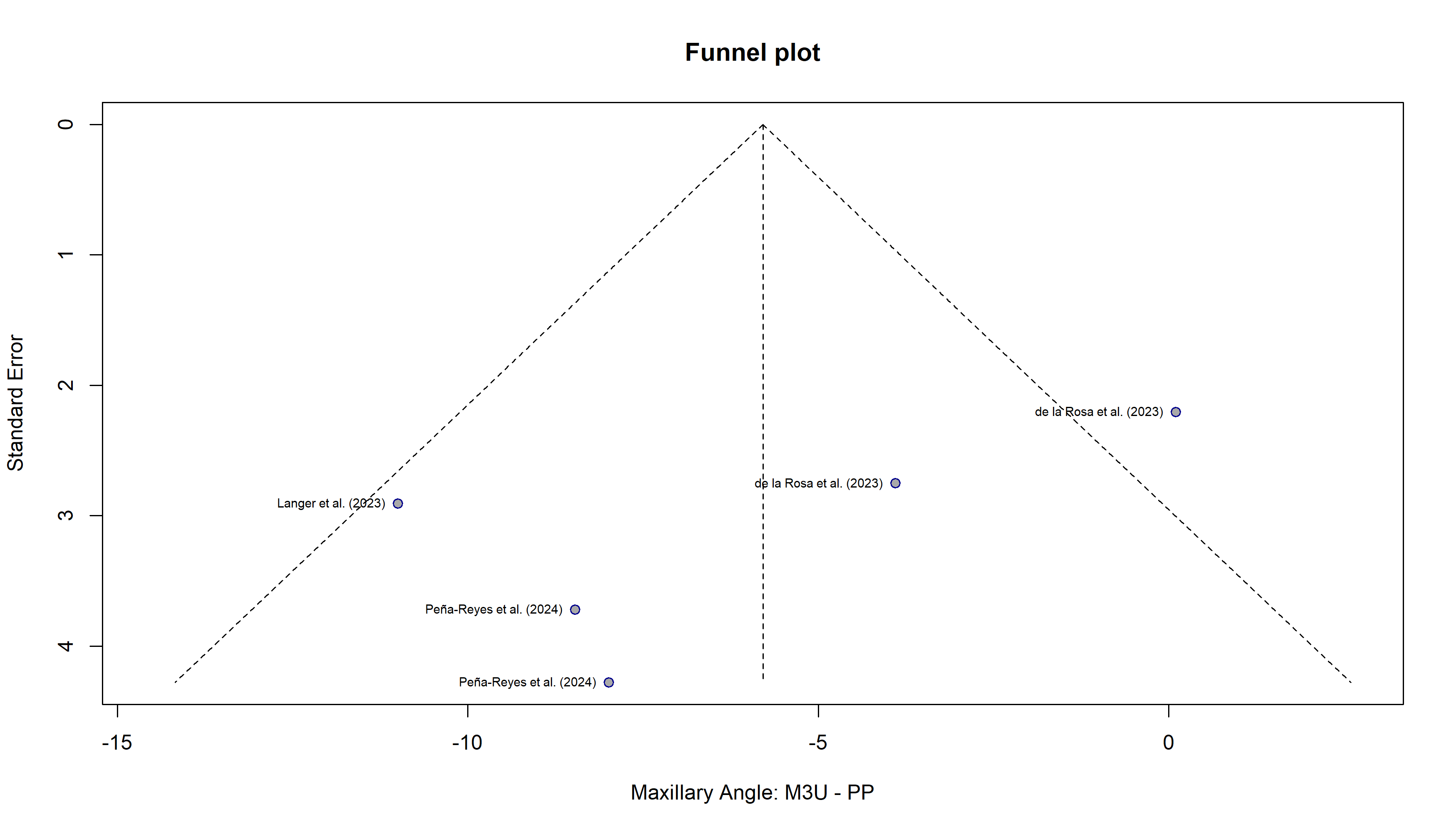

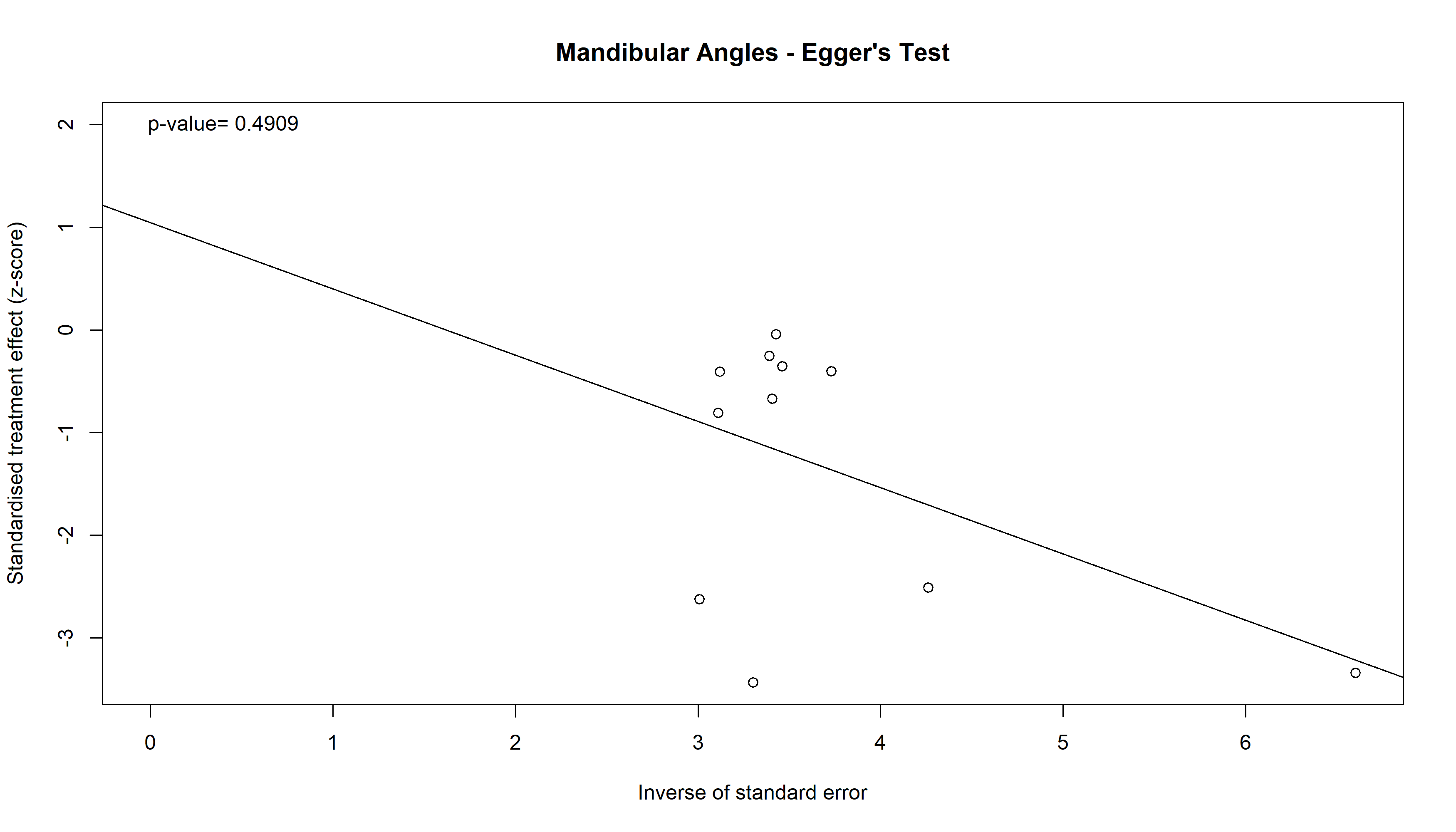

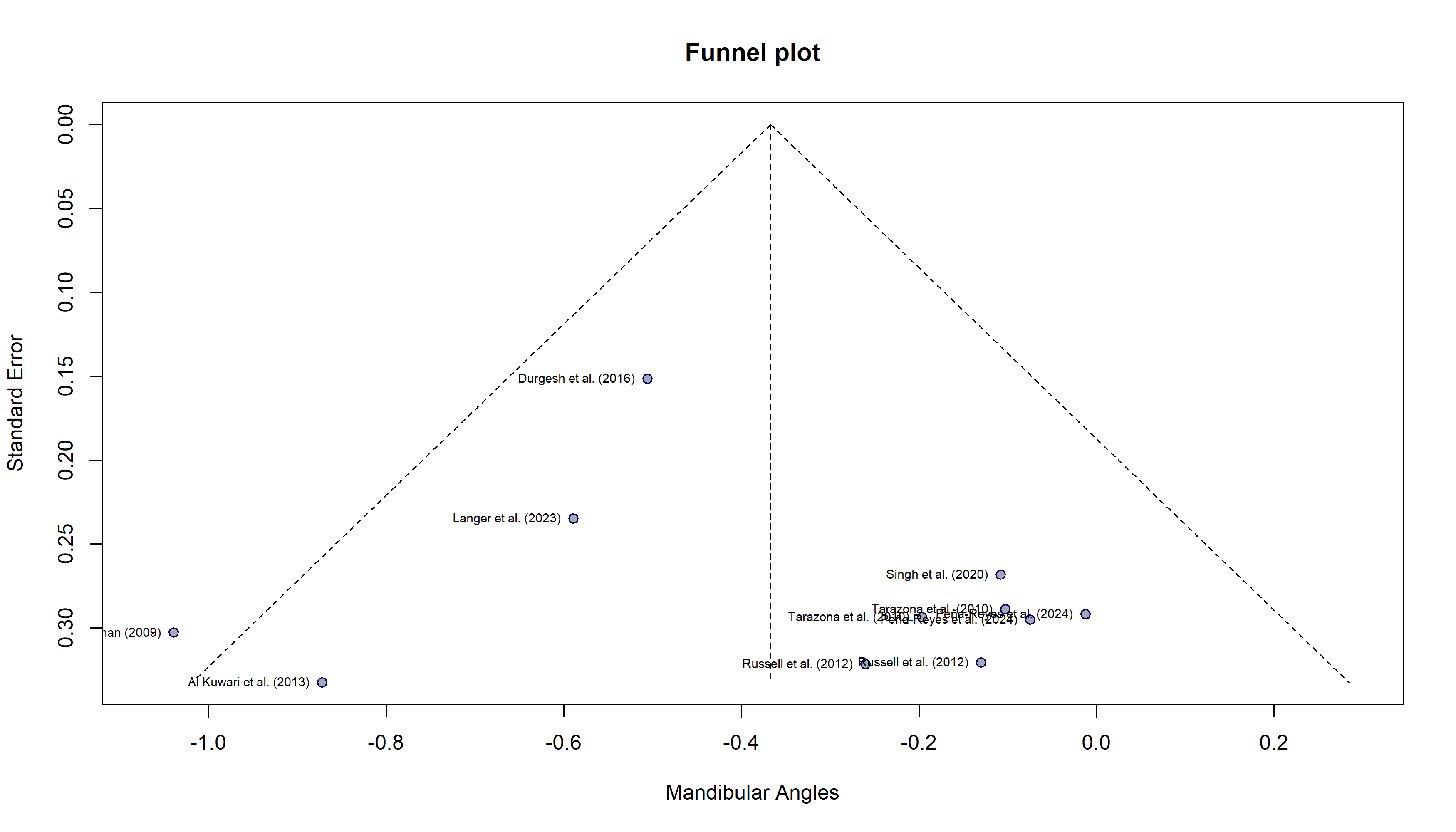

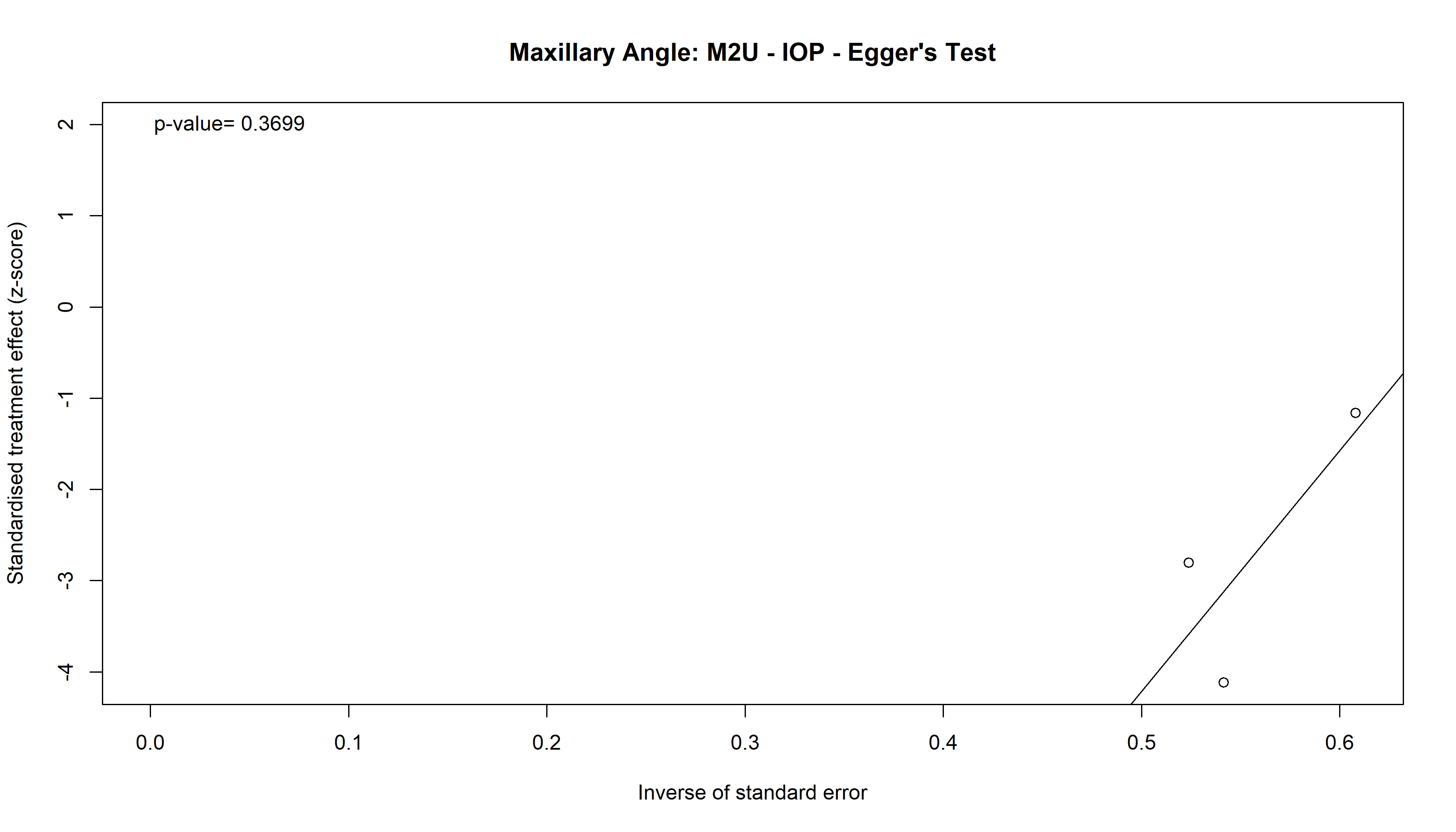
**

**Figure S3:**

**
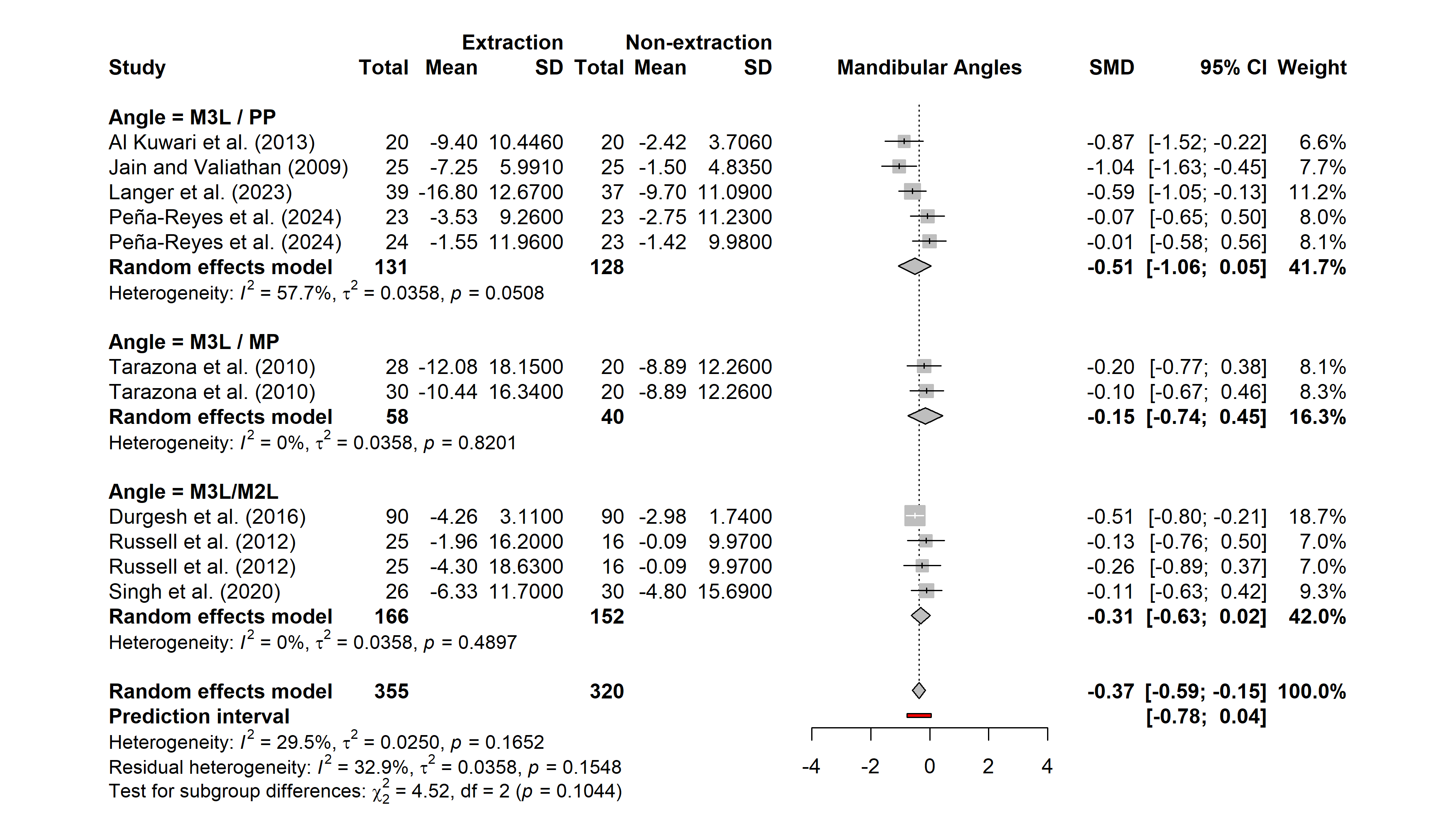
**

**Appendix S1.**

| **Section and Topic** | **Item #** | **Checklist item** | **Reported (Yes/No)** |
| --- | --- | --- | --- |
| **TITLE** | | |  |
| Title | 1 | Identify the report as a systematic review. | Yes |
| **BACKGROUND** | | |  |
| Objectives | 2 | Provide an explicit statement of the main objective(s) or question(s) the review addresses. | Yes |
| **METHODS** | | |  |
| Eligibility criteria | 3 | Specify the inclusion and exclusion criteria for the review. | Yes |
| Information sources | 4 | Specify the information sources (e.g. databases, registers) used to identify studies and the date when each was last searched. | Yes |
| Risk of bias | 5 | Specify the methods used to assess risk of bias in the included studies. | Yes |
| Synthesis of results | 6 | Specify the methods used to present and synthesise results. | Yes |
| **RESULTS** | | |  |
| Included studies | 7 | Give the total number of included studies and participants and summarise relevant characteristics of studies. | Yes |
| Synthesis of results | 8 | Present results for main outcomes, preferably indicating the number of included studies and participants for each. If meta-analysis was done, report the summary estimate and confidence/credible interval. If comparing groups, indicate the direction of the effect (i.e. which group is favoured). | Yes |
| **DISCUSSION** | | |  |
| Limitations of evidence | 9 | Provide a brief summary of the limitations of the evidence included in the review (e.g. study risk of bias, inconsistency and imprecision). | Yes |
| Interpretation | 10 | Provide a general interpretation of the results and important implications. | Yes |
| **OTHER** | | |  |
| Funding | 11 | Specify the primary source of funding for the review. | Yes |
| Registration | 12 | Provide the register name and registration number. | Yes |

*From:*  Page MJ, McKenzie JE, Bossuyt PM, Boutron I, Hoffmann TC, Mulrow CD, et al. The PRISMA 2020 statement: an updated guideline for reporting systematic reviews. BMJ 2021;372:n71. doi: 10.1136/bmj.n71. This work is licensed under CC BY 4.0. To view a copy of this license, visit <https://creativecommons.org/licenses/by/4.0/>

| **Section and Topic** | **Item #** | **Checklist item** | **Location where item is reported** |
| --- | --- | --- | --- |
| **TITLE** | | |  |
| Title | 1 | Identify the report as a systematic review. | Page 1 |
| **ABSTRACT** | | |  |
| Abstract | 2 | See the PRISMA 2020 for Abstracts checklist. | Page 1-2 |
| **INTRODUCTION** | | |  |
| Rationale | 3 | Describe the rationale for the review in the context of existing knowledge. | Page 2-3 |
| Objectives | 4 | Provide an explicit statement of the objective(s) or question(s) the review addresses. | Page 3 |
| **METHODS** | | |  |
| Eligibility criteria | 5 | Specify the inclusion and exclusion criteria for the review and how studies were grouped for the syntheses. | Page 4-5 |
| Information sources | 6 | Specify all databases, registers, websites, organisations, reference lists and other sources searched or consulted to identify studies. Specify the date when each source was last searched or consulted. | Page 4 |
| Search strategy | 7 | Present the full search strategies for all databases, registers and websites, including any filters and limits used. | Appendix S2 |
| Selection process | 8 | Specify the methods used to decide whether a study met the inclusion criteria of the review, including how many reviewers screened each record and each report retrieved, whether they worked independently, and if applicable, details of automation tools used in the process. | Page 5-6 |
| Data collection process | 9 | Specify the methods used to collect data from reports, including how many reviewers collected data from each report, whether they worked independently, any processes for obtaining or confirming data from study investigators, and if applicable, details of automation tools used in the process. | Page 5-6 |
| Data items | 10a | List and define all outcomes for which data were sought. Specify whether all results that were compatible with each outcome domain in each study were sought (e.g. for all measures, time points, analyses), and if not, the methods used to decide which results to collect. | Page 5-6 |
|  | 10b | List and define all other variables for which data were sought (e.g. participant and intervention characteristics, funding sources). Describe any assumptions made about any missing or unclear information. | Page 5 |
| Study risk of bias assessment | 11 | Specify the methods used to assess risk of bias in the included studies, including details of the tool(s) used, how many reviewers assessed each study and whether they worked independently, and if applicable, details of automation tools used in the process. | Page 5-6, Table 1 |
| Effect measures | 12 | Specify for each outcome the effect measure(s) (e.g. risk ratio, mean difference) used in the synthesis or presentation of results. | Page 6-7 |
| Synthesis methods | 13a | Describe the processes used to decide which studies were eligible for each synthesis (e.g. tabulating the study intervention characteristics and comparing against the planned groups for each synthesis (item #5)). | Page 6 |
|  | 13b | Describe any methods required to prepare the data for presentation or synthesis, such as handling of missing summary statistics, or data conversions. | Page 7 |
|  | 13c | Describe any methods used to tabulate or visually display results of individual studies and syntheses. | Page 6, Table 3, Figure 4,5 |
|  | 13d | Describe any methods used to synthesize results and provide a rationale for the choice(s). If meta-analysis was performed, describe the model(s), method(s) to identify the presence and extent of statistical heterogeneity, and software package(s) used. | Page 6-7 |
|  | 13e | Describe any methods used to explore possible causes of heterogeneity among study results (e.g. subgroup analysis, meta-regression). | Page 8-9 |
|  | 13f | Describe any sensitivity analyses conducted to assess robustness of the synthesized results. | Page 9-10 |
| Reporting bias assessment | 14 | Describe any methods used to assess risk of bias due to missing results in a synthesis (arising from reporting biases). | Page 10 |
| Certainty assessment | 15 | Describe any methods used to assess certainty (or confidence) in the body of evidence for an outcome. | Page 10 |
| **RESULTS** | | |  |
| Study selection | 16a | Describe the results of the search and selection process, from the number of records identified in the search to the number of studies included in the review, ideally using a flow diagram. | Page 10, Figure 2 |
|  | 16b | Cite studies that might appear to meet the inclusion criteria, but which were excluded, and explain why they were excluded. | Page 10 |
| Study characteristics | 17 | Cite each included study and present its characteristics. | Page 11, Table 2 |
| Risk of bias in studies | 18 | Present assessments of risk of bias for each included study. | Page 12, Figure 3 |
| Results of individual studies | 19 | For all outcomes, present, for each study: (a) summary statistics for each group (where appropriate) and (b) an effect estimate and its precision (e.g. confidence/credible interval), ideally using structured tables or plots. | Page 13-14, Table 3 |
| Results of syntheses | 20a | For each synthesis, briefly summarise the characteristics and risk of bias among contributing studies. | Page 13 |
|  | 20b | Present results of all statistical syntheses conducted. If meta-analysis was done, present for each the summary estimate and its precision (e.g. confidence/credible interval) and measures of statistical heterogeneity. If comparing groups, describe the direction of the effect. | Page 13-14 |
|  | 20c | Present results of all investigations of possible causes of heterogeneity among study results. | Page 8-9 |
|  | 20d | Present results of all sensitivity analyses conducted to assess the robustness of the synthesized results. | Page 9-10 |
| Reporting biases | 21 | Present assessments of risk of bias due to missing results (arising from reporting biases) for each synthesis assessed. | Page 10 |
| Certainty of evidence | 22 | Present assessments of certainty (or confidence) in the body of evidence for each outcome assessed. | Page 10 |
| **DISCUSSION** | | |  |
| Discussion | 23a | Provide a general interpretation of the results in the context of other evidence. | Page 11-12 |
|  | 23b | Discuss any limitations of the evidence included in the review. | Page 13 |
|  | 23c | Discuss any limitations of the review processes used. | Page 13 |
|  | 23d | Discuss implications of the results for practice, policy, and future research. | Page 13-14 |
| **OTHER INFORMATION** | | |  |
| Registration and protocol | 24a | Provide registration information for the review, including register name and registration number, or state that the review was not registered. | Page 4, Prospero |
|  | 24b | Indicate where the review protocol can be accessed, or state that a protocol was not prepared. | Page 4 |
|  | 24c | Describe and explain any amendments to information provided at registration or in the protocol. | Not Applicable |
| Support | 25 | Describe sources of financial or non-financial support for the review, and the role of the funders or sponsors in the review. | Funding section |
| Competing interests | 26 | Declare any competing interests of review authors. | Competing interests |
| Availability of data, code and other materials | 27 | Report which of the following are publicly available and where they can be found: template data collection forms; data extracted from included studies; data used for all analyses; analytic code; any other materials used in the review. | Availability of data and material |

*From:*  Page MJ, McKenzie JE, Bossuyt PM, Boutron I, Hoffmann TC, Mulrow CD, et al. The PRISMA 2020 statement: an updated guideline for reporting systematic reviews. BMJ 2021;372:n71. doi: 10.1136/bmj.n71. This work is licensed under CC BY 4.0. To view a copy of this license, visit <https://creativecommons.org/licenses/by/4.0/>

**Appendix S2. Search strategies used in each electronic database.**

## **PUBMED**

("orthodontics"[MeSH Terms] OR "orthodontic treatment"[All Fields]) AND ("third molar angulation"[All Fields] OR "angulation"[All Fields]) AND ("teeth extraction"[MeSH Terms] OR "orthodontic extraction"[All Fields])

Filters applied: Humans, Publication dates from 2000/01/01 to 2024/11/30
Last search date: November 30, 2024

## **SCOPUS**

TITLE-ABS-KEY("orthodontics" OR "orthodontic treatment") AND TITLE-ABS-KEY("third molar angulation" OR "angulation") AND TITLE-ABS-KEY("teeth extraction" OR "orthodontic extraction")

LIMIT-TO(PUBYEAR, 2000–2024)
Last search date: November 30, 2024

**Appendix S3. Additional details on the review methodology**

**Data synthesis**

For all included studies, the change in third molar angulation between pre-treatment (T0) and post-treatment (T1) was extracted for both extraction and non-extraction groups. These changes were synthesized using either Mean Differences (MD) or Standardized Mean Differences (SMD), depending on the heterogeneity of outcome measurements and scales used across studies.

The SMD was applied when angular measurements were reported in different planes or reference systems, allowing for comparability between studies. The conventional thresholds of 0.2, 0.5, and 0.8 were used to interpret small, moderate, and large effects, respectively, following Schünemann et al. (2008).

For outcomes consistently measured in degrees (e.g., M3U/PP, M3L/MP), pooled MDs were used. Heterogeneity was assessed using the I² statistic and Cochrane’s Q test, and random-effects models were applied to account for between-study variability.

Subgroup analyses were also performed to evaluate whether the type of angulation (e.g., M3U/M2U vs. M3U/IOP) influenced the direction or magnitude of the effect.

**References to Appendix S3**

Norman GR, Sloan JA, Wyrwich KW. The truly remarkable universality of half a standard deviation: confirmation through another look. Expert Rev Pharmacoecon Outcomes Res 2004; 4: 581–585.

Schünemann H, Brozek J, Oxman A, eds. GRADE handbook for grading quality of evidence and strength of recommendation. Version 3.2. The GRADE Working Group; 2009. [updated March 2009] http://www.who.int/hiv/topics/mtct/grade_handbook.pdf.
